# The Effectiveness of Crisis Line Services: A Systematic Review

**DOI:** 10.3389/fpubh.2019.00399

**Published:** 2020-01-17

**Authors:** Adam S. Hoffberg, Kelly A. Stearns-Yoder, Lisa A. Brenner

**Affiliations:** ^1^Department of Veterans Affairs, Rocky Mountain Mental Illness, Research, Education and Clinical Center, Aurora, CO, United States; ^2^Department of Physical Medicine and Rehabilitation, University of Colorado, Anschutz Medical Campus, Aurora, CO, United States; ^3^Departments of Psychiatry and Neurology, University of Colorado, Anschutz Medical Campus, Aurora, CO, United States

**Keywords:** systematic review, crisis line, suicide, health services, self-directed violence, prevention, public health, quality of care

## Abstract

**Background:** Crisis lines are a standard component of a public health approach to suicide prevention. Clinical aims include reducing individuals' crisis states, psychological distress, and risk of suicide. Efforts may also include enhancing access and facilitating connections to behavioral health care. This review examines models of crisis line services for demonstrated effectiveness.

**Methods:** Literature searches of Medline, EMBASE, PsycINFO, Web of Science, CINAHL, Cochrane Library, and Google Scholar were conducted from January 1, 1990, to May 7, 2018. Experts were contacted, and references were mined for additional studies. Eligible studies provided health- or utilization-related effectiveness outcome(s). Results were graded according to the Oxford Centre for Evidence-Based Medicine and evaluated for risk of bias using the Effective Public Health Practice Project quality assessment tool for quantitative studies.

**Results:** Thirty-three studies yielded effectiveness outcomes. In most cases findings regarding crisis calls vs. other modalities were presented. Evaluation approaches included user- and helper-reported data, silent monitoring, and analyses of administrative records. About half of studies reported immediate proximal outcomes (during the crisis service), and the remaining reported distal outcomes (up to four years post-contact). Most studies were rated at Oxford level four evidence and 80% were assessed at high risk of bias.

**Conclusions:** High quality evidence demonstrating crisis line effectiveness is lacking. Moreover, most approaches to demonstrating impact only measured proximal outcomes. Research should focus on innovative strategies to assess proximal and distal outcomes, with a specific focus on behavioral health treatment engagement and future self-directed violence.

## Introduction

### Rationale

In the United States (US), from 1999 through 2017, the age-adjusted suicide rate increased 33% from 10.5 to 14.0 per 100,000 (1) and worldwide suicide remains a pressing concern. Upstream efforts to prevent suicide include crisis line services (e.g., call, chat, text). During such interactions, responders address the crisis at hand with the aim of reducing crisis states, psychological distress, and risk of suicide. This may include facilitating evaluation of imminent risk by local first responders. In addition, within the context of a crisis line contact, responders may provide resources and strategies to facilitate treatment referrals and engagement in care. Given the key role of crisis lines within a comprehensive public health strategy for suicide prevention, it is critical to know whether they are meeting their intended goals. The primary goal of crisis line effectiveness research is to evaluate the immediate proximal and/or longer-term distal effect(s) of such interventions. These effects may be measured using a wide-range of outcomes, including health- and service use-related client outcomes data regarding prevention of self-directed violence, enhanced mood, satisfaction, compliance with responder interventions, and/or service utilization, as well as outcomes regarding responder responses, such as intervention style and referral recommendations.

### Objectives

The purpose of this systematic review is to establish the state of the science on crisis line effectiveness research. This review provides an exhaustive account of published literature, identifying not only strengths and biases present in the evidence, but also gaps, limitations, and future research opportunities. Within this framework, we specifically examined the literature to identify and appraise: (1) immediate proximal as well as longer-term distal outcomes measuring crisis line effects; (2) data collection approaches utilized to measure impact; and (3) study design and risks of bias informing the strength of current evidence.

### Research Question

The key question (KQ) of interest inquired whether there are models of service delivery (crisis line phone, chat, or text) with demonstrated effectiveness.

## Methods

### Study Design

This systematic review was conducted in accordance with Preferred Reporting Items for Systematic Reviews and Meta-Analyses (PRISMA) guidelines (2). A completed PRISMA Checklist is available (See Supplementary Table 3).

### Participants, Interventions, Comparators

For the PRISMA screening and eligibility stages, each study was assessed independently by two reviewers (LAB, KSY) and a third reviewer (ASH) resolved disagreements. Eligibility Criteria were defined according to the PICO(TS) framework: (2, 3)

- Population—Crisis line users consisted of any age.
- Intervention/Exposure—Use of crisis line phone, chat, or text services (see Supplementary Table 2). An intervention was not required for inclusion (e.g., surveys or administrative data were included). However, studies were excluded if they only provided results on demographic profiles of crisis line utilizers without effectiveness outcomes as described below.
- Comparison—Not required for inclusion.
- Outcomes—All health- and use-related effectiveness outcomes both immediate proximal and longer-term distal, including SDV, client mood, satisfaction, compliance, and service utilization, as well as responder responses (e.g., referrals, intervention styles) (see Supplementary Table 2).
- Timing/Setting—Restrictions were not based on timing, setting, or study design.

Only studies including original data and published in a peer-reviewed journal from January 1, 1990 through May 7, 2018 were included.

### Systematic Review Protocol

A protocol for this review is registered in PROSPERO International prospective register of systematic reviews under registration number CRD42019127249 (4).

### Search Strategy

Databases were searched using controlled subject heading vocabulary and key words for suicidal self-directed violence (SDV), (5) combined with controlled subject heading vocabulary and key words for crisis line services (see Supplementary Table 1). Searches were limited to English language only. Experts were contacted, and references were mined for additional studies. Complete references were exported from each literature source into EndNote X8, duplicates were removed, and the remaining entries were imported into Covidence review software.

### Data Sources, Studies Sections, and Data Extraction

The final literature search of OVID Medline, EMBASE, OVID PsycINFO, Web of Science, CINAHL, Cochrane Library, and Google Scholar was conducted on May 7, 2018. Data from included articles were abstracted into evidence tables by two authors (ASH, KSY; conflicts resolved via discussion with LAB). Extracted data from each article included a description of the crisis line service, sample characteristics, study time period, effectiveness domains measured, source of outcome measurements, proximity of outcome measurement to the intervention, and effectiveness findings (see Supplementary Table 4).

Included studies were independently evaluated by two reviewers (ASH, KSY) in a custom Research Electronic Data Capture (REDCap) database (6) with disagreements resolved by consensus discussion with a third reviewer (LAB).

### Strength of Evidence and Risk of Bias

Included studies were graded by level of evidence according to the Oxford Centre for Evidence-Based Medicine (7) (See Table 1). In some cases, “higher level” Oxford grades from well-designed and executed observational studies provided stronger evidence (lower risk of bias) than “lower level” Oxford graded randomized controlled trials (RCT) with extensive biases. To address this limitation and complement the Oxford quality ratings, risk of bias was also assessed independently by two raters using the Effective Public Health Practice Project (EPHPP) quality assessment tool for quantitative studies (41). The EPHPP assessment was conducted in a custom REDCap database. The EPHPP tool bias items included selection bias, study design, confounders, blinding, data collection, withdrawals/dropouts, and other sources (e.g., no disclosure of conflicts of interest) (41). To inform the study design appraisal, included studies were classified by study design using the Taxonomy of Study Design Tool (42). Guidance for bias ratings was drawn from the EPHPP data dictionary, and summarized as follows: Selection bias considered to what extent study participants were likely to be representative of the target population, as well as the proportion of selected individuals who agreed to participate in the study; Study design considered the likelihood of bias in the allocation process for experimental designs, and for observational designs, the extent that assessments of exposure and outcome are likely to be independent; Confounding examined to what extent important variables were controlled for in the study design (by matching or stratification), and/or in the analyses; Blinding assessed detection and reporting bias, such as whether the assessors were aware of the research condition and/or the participants were aware of the research question(s); Data collection methods were rated on the validity, reliability and use of standardized outcome measures, including distinctions between self-reported data, objective data retrieved by investigators, and extracted data from administrative records; Withdrawals and drop-outs assessed the proportion of participants remaining in the study through the final data collection period (if applicable); and Other sources of bias included intervention integrity and utilizing appropriate analyses for the research questions (43). Each of these domains, if applicable, was rated as having a low, moderate, or high risk of bias based on these standard guidelines. An overall risk of bias rating was then generated (43, 44). Ratings were based only on information reported in the study. All discrepancies were discussed until reviewers reached consensus regarding the extent of bias present in each domain and overall.

**Table 1:** Design, sources of bias, overall bias, and oxford quality rating by study.

**Study**	**Study design**	**Source of bias**						**Overall bias**	**Oxford quality rating**
		**Selection bias**	**Study design**	**Confounders**	**Blinding**	**Data collection**	**Withdrawals/ dropouts**		
de Anda and Smith (8)	Cross-sectional	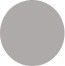	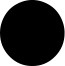	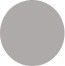	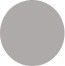	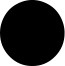	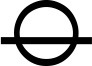	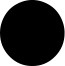	4
Daigle and Mishara (9)	Cross-sectional	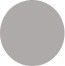	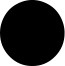	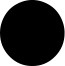	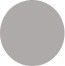	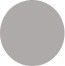	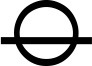	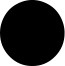	4
Jianlin (10)	Before-after	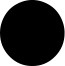	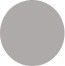	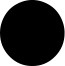	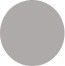	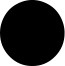	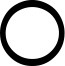	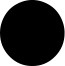	4
Leenaars and Lester (11)	Retrospective cohort	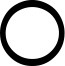	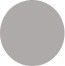	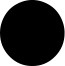	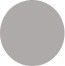	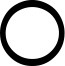	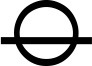	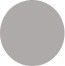	3
Mishara and Daigle (12)	Before-after	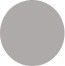	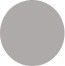	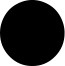	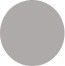	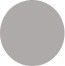	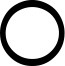	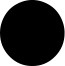	4
King et al. (13)	Before-after	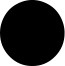	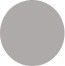	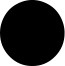	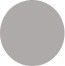	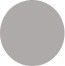	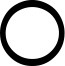	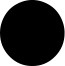	4
Leenaars and Lester, Study 1 (14)	Cross-sectional	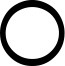	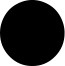	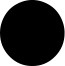	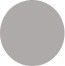	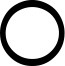	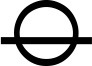	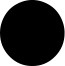	4
Leenaars and Lester, Study 2 (14)	Retrospective cohort	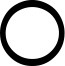	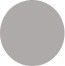	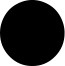	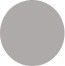	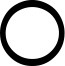	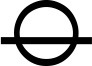	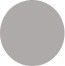	3
Mishara et al. (15)	RCT	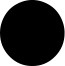	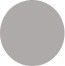	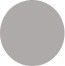	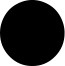	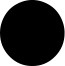	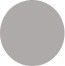	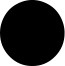	1
Latzer and Gilat (16)	Cross-sectional	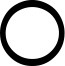	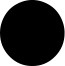	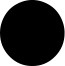	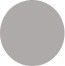	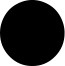	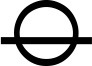	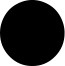	4
Gould et al. (17)	Before-after	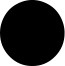	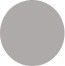	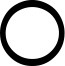	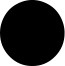	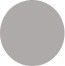	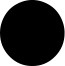	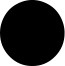	4
Kalafat et al. (18)	Before-after	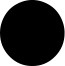	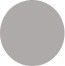	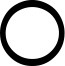	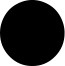	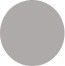	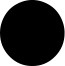	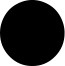	4
Mishara et al. (19)	Cross-sectional	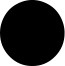	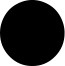	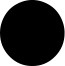	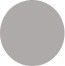	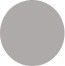	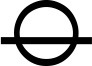	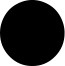	4
Mishara et al. (20)	Before-after	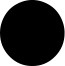	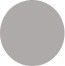	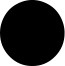	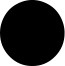	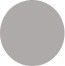	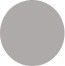	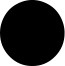	4
Fukkink and Hermanns (21)	Controlled before-after	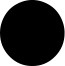	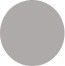	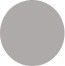	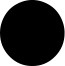	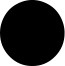	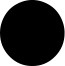	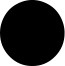	4
Witte et al. (22)	Before-after	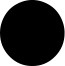	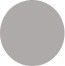	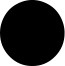	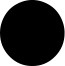	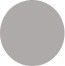	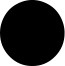	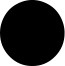	4
Chavan et al. (23)	Cross-sectional	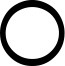	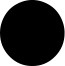	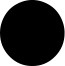	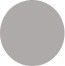	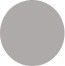	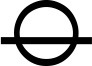	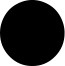	4
Coveney et al. (24)	Cross-sectional	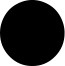	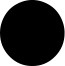	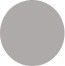	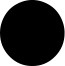	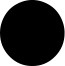	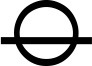	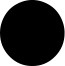	4
Gould et al. (25)	Cross-sectional	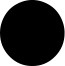	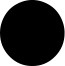	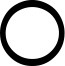	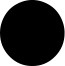	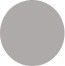	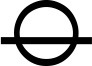	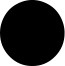	4
Knox et al. (26)	Cross-sectional	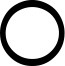	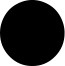	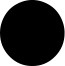	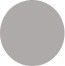	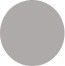	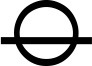	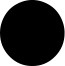	4
Tan et al. (27)	Cross-sectional	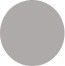	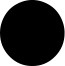	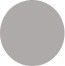	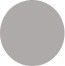	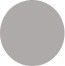	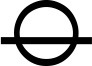	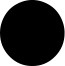	4
Britton et al. (28)	Cross-sectional	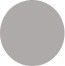	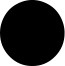	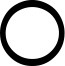	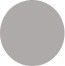	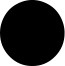	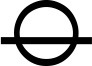	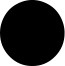	4
Gould et al. (29)	RCT	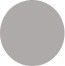	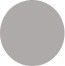	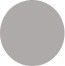	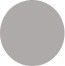	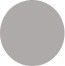	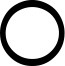	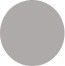	1
Pil et al. (30)	N/A	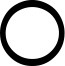	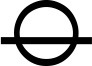	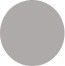	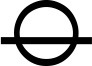	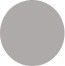	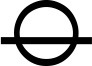	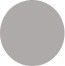	4
Britton et al. (31)	Retrospective cohort	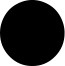	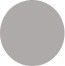	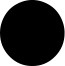	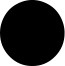	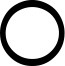	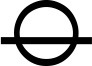	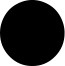	3
Gould et al. (32)	Cross-sectional	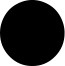	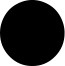	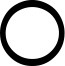	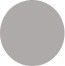	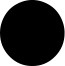	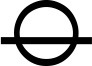	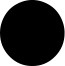	4
Mishara et al., Study 1 (33)	Cross-sectional	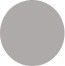	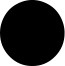	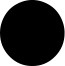	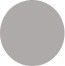	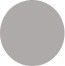	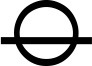	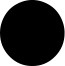	4
Mishara et al., Study 2 (33)	Before-after	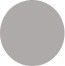	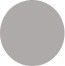	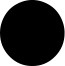	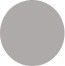	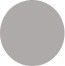	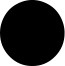	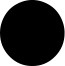	4
Tyson et al. (34)	Before-after	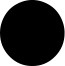	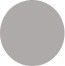	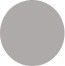	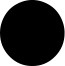	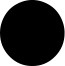	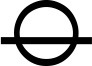	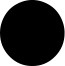	4
Mokkenstorm et al. (35)	Before-after	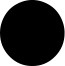	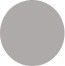	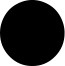	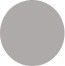	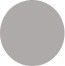	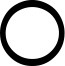	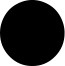	4
Ramchand et al. (36)	Controlled before-after	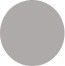	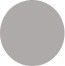	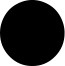	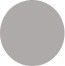	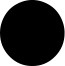	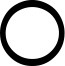	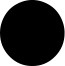	4
Rasmussen et al. (37)	Cross-sectional	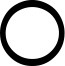	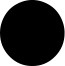	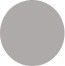	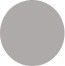	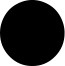	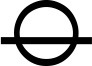	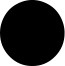	4
Chan et al. (38)	Retrospective cohort	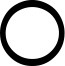	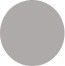	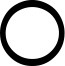	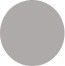	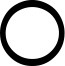	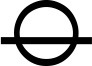	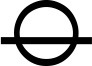	3
Gould et al. (39)	Before-after	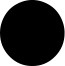	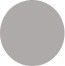	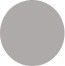	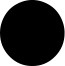	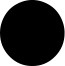	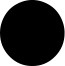	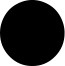	4
Mejias-Martin et al. (40)	Cross-sectional	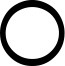	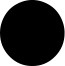	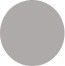	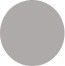	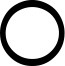	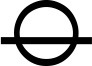	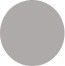	4

*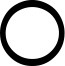, low; 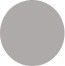, moderate; 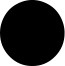, high; 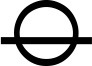, not applicable; RCT, Randomized Controlled Trial*.

### Data Analysis

Variability of study designs and outcome measurement precluded a meta-analytic approach to synthesis. Findings were not quantitatively synthesized because included studies were mostly a mix of observational and quasi-experimental design, and often utilized unstandardized measurement approaches to assess a variety of outcomes across many effectiveness domains. Therefore, a descriptive synthesis approach was utilized.

## Results

### Study Selection and Characteristics

**Figure 1:**
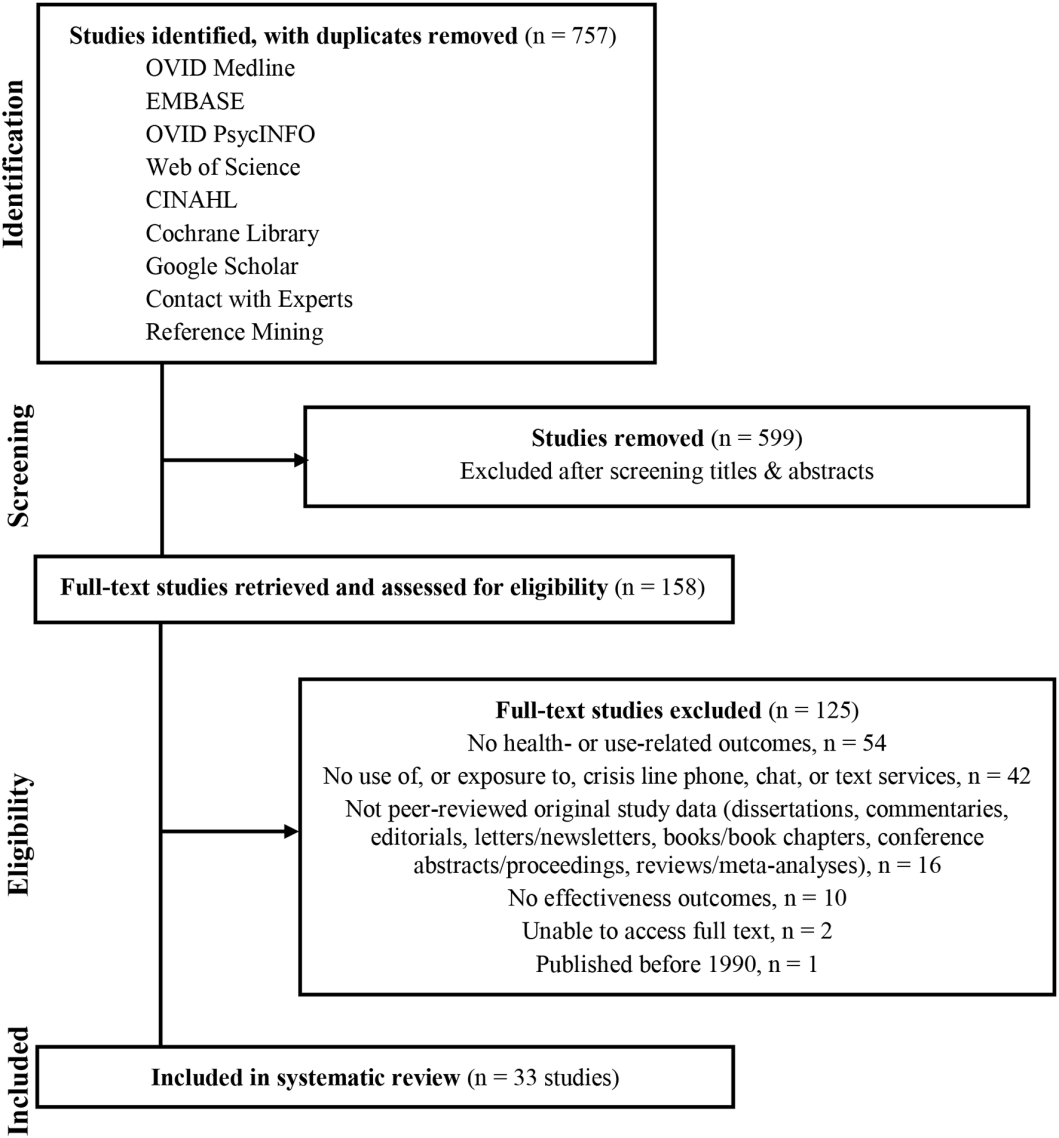
PRISMA literature flow diagram.

Of the 757 studies screened, 33 met eligibility criteria and were included in the review (See Figure 1). Whereas the vast majority of studies described outcome data measured from crisis calls, three included effectiveness outcomes from crisis chat (21, 30, 35). No studies examined crisis line text outcomes. Crisis line call centers included in the review were staffed by a range of responders (e.g., volunteers, paid employees). Approaches to effectiveness outcome measurement also varied, and included user- and responder-reported outcomes, ratings by silent monitors unobtrusively observing crisis line calls, and coding of administrative records (e.g., from clinical forms, chat logs, and call recordings) (see Supplementary Tables 2, 4).

### Synthesized Findings and Risk of Bias

The overall risk of bias of included studies was high, and the most frequent Oxford level of evidence was four. Only one study identified was low risk of bias, five studies were rated moderate risk of bias, and the remaining were high risk of bias (See Table 1). There were many common sources of bias found in the moderate and high risk of bias studies. Specifically, selection bias was highly prevalent (e.g., many studies excluded crisis line users with the highest [imminent] suicide risk and also inconsistently approached crisis line users for participation). The vast majority of included studies had risks of bias in confounding in the study design and/or analyses, leading to challenges in interpreting potentially spurious associations or findings that could be related to a variable other than the crisis intervention. Furthermore, data collection, measurement, and detection biases (e.g., using unblinded approaches and tools not shown to be valid), as well as attrition bias (e.g., when measuring distal outcomes) contributed to downgraded ratings in the strength of the evidence. Based on these appraisals, the overall strength of evidence for outcomes measuring crisis line effectiveness was determined to be low.

### Study Design

Only two studies were RCTs [Gould et al. (29) moderate risk of bias and Mishara et al. (15) high risk of bias; Oxford quality ratings of 1]. Additionally, there were four cohort studies (11, 14, 31, 38), and the remaining studies were a mix of observational and quasi-experimental design, most of which were cross-sectional or single group before-after designs (high risk of bias; Oxford quality ratings of 4).

### Immediate Proximal Evidence of Effectiveness

In about half of studies (16 studies) immediate proximal outcomes during and/or at the end of a crisis line service were evaluated (See Supplementary Table 4). Immediate proximal evidence consisted almost exclusively of cross-sectional studies of a single measurement timepoint or single-group before-after study designs measuring change from the beginning (pre-) to the end (post-) of the crisis line intervention; one proximal RCT was noted. For the most part, adolescents and adults utilized the crisis lines services evaluated, however there were five adult only samples, which included three US Veteran studies, and four studies in which age was not reported. Regarding location of crisis lines evaluated, eight studies were from the US, two from the United Kingdom (UK), and one each from Australia, Israel, Canada, Amsterdam, and Spain. Proximal outcomes measured included client mood/satisfaction at the end of the call or change from the beginning to the end of the call (nine studies), helper responses/approaches used during the call (eight studies), the provision of referrals (seven studies), and changes in SDV such as suicidal thoughts (four studies).

Approaches to outcome measurement also varied, including six studies that utilized silent monitors or call/chat log ratings that were shown to be reliable, and three studies incorporated validated assessment tools. However, seven studies relied on administrative/clinical records such as routine call sheets completed by responders, and five studies used approaches not shown to be reliable or valid. Note some studies measured effectiveness across more than one domain and using a mixture of validated and unvalidated approaches.

Notable immediate proximal evidence included a before-after study by King et al. (13) (high risk of bias; Oxford quality rating of 4) in which 100 taped calls between March 1998 and March 1999 to the Kids HelpLine in Australia were analyzed. Callers were assessed for suicidal ideation, intent, and mental state using a mixture of standardized and unstandardized approaches, including assessment items adopted from the Mini-International Neuropsychiatric Interview (MINI) Modules A and C (45). Two independent raters analyzed each tape for changes and identified a significant decrease in suicidal ideation and improvement in mental state from the beginning to the end of a call (both *p* < 0.0005). A substantial decrease in the proportion of callers rated imminent risk at the end of calls was also noted. However, 14% of callers remained suicidal at the end of the call (13).

In another study employing reliable independent raters and the use of a validated assessment tool, Mishara and colleagues (19, 20) (high risk of bias; Oxford quality rating of 4) analyzed 1,431 adults crisis calls to the Hopeline Network in the US between August 2003 and May 2004. Caller mood/states and helper responses were evaluated via ratings by two silent monitors observing unobtrusively, and differences between centers were evaluated by the Crisis Call Outcome Rating Scale (CCORS) (46). Reliability analyses were performed for silent monitor observations of helpers, and interrater agreement was found to be quite high throughout (19, 20). They found an overall positive mean effect (*p* < 0.001), but many variables did not significantly change from the beginning to the end of the call according to ratings by the silent observers (19, 20). Responder approaches were found to impact caller outcomes, with a supportive approach and good contact associated with positive mood/state changes (*p* < 0.001). Furthermore, there was significant variability in effectiveness across call centers, measured via the CCORS (*p* < 0.03), and a supportive, collaborative approach with good contact, empathy, and respect were all associated with higher CCORS scores and fewer hang ups (all *p* < 0.001) (19, 20). The authors also noted that 50.5% of callers were not asked about suicidal ideation, and responders failed to meet minimum acceptability standards in 15.6% of calls, including lacking empathy, respect, poor initial contact, and stunningly in four cases the helper told the caller to go ahead and kill himself (19, 20). In two cases, an emergency rescue protocol was initiated by the research team when the helper failed to do so with callers at imminent risk.

A more recent example of a reliable silent monitoring approach was conducted by Ramchand et al. (36) (high risk of bias; Oxford quality rating of 4) in which 241 calls from 10 American Association of Suicidology-accredited hotlines in California during the Spring and Summer of 2014 were monitored. The protocol was developed from existing work by Gould and colleagues (25, 29) and included use of the Lifeline Quality Improvement Monitoring Tool. Monitors identified a mean 43% decreased caller distress from beginning to end of call (range 28–64%), with decreased distress associated with the crisis center NSPL network membership (Odds Ratio [OR] 2.72; *p* = 0.024) (36). Responders at NSPL centers were also more likely to ask about current suicide ideation (77 vs. 52%; OR 3.6; *p* < 0.01), recent ideation (31 vs. 16%; OR 2.5; *p* = 0.02), and past attempts (27 vs. 10%; OR 3.7; *p* < 0.01) (36). The researchers also noted that a mean of 7% of all calls were put on hold (range 0–26% across centers) (36).

In terms of crisis services provided to Veterans, Knox et al. (high risk of bias, Oxford quality rating of 4) analyzed the implementation and early utilization of the Veterans Crisis Line (VCL). Between July 2007 and September 2010, 171,000 calls were made to the VCL. Effectiveness was analyzed via responder referrals to either Suicide Prevention Coordinators (SPC) and/or other Veterans Health Administration (VHA) and community programs. From VCL inception in July 2007 through 2008, approximately 4,000 referrals were made to SPCs, and this increased to 16,000 total referrals by the end of September 2010.

Also of note, in a cross-sectional study of VCL, Britton et al. (high risk of bias; Oxford quality rating of 4) analyzed 646 calls during a 1 week period in 2010 to ascertain responder referral actions at the end of each call (28). Results indicated that 84% of calls ended with a favorable outcome, defined as either resolution during the call or referral to a local health care provider, with the remaining 16% classified as unresolved/declined referral. In the univariate analysis, higher risk callers had significantly higher odds of the call ending in a referral (77 vs. 49%; Relative Risk Ratio [RRR] 2.70; 95% Confidence Interval [CI] 1.64–4.47), and in the multivariate analysis callers at higher risk approached significance in more calls resolved vs. unresolved compared with lower risk callers (RRR 0.56; 95% CI 0.30–1.04; *p* = 0.067). For 54% of callers, responders provided reasons for determining higher vs. lower risk callers, and reported these judgments were based on intent to die (OR 8.47; 95% CI 3.85–18.63) and absence of future plans (OR 10.45; 95% CI 2.84–38.40) (28).

In the most robust proximal evidence of effectiveness, Gould et al. (29) conducted the first national RCT to evaluate the immediate proximal effect of a crisis center intervention and training strategy using a dynamic wait-listed roll-out design across the US National Suicide Prevention Lifeline (NSPL) network of crisis hotline centers. Effectiveness was measured by silent monitoring of 1,507 calls between June 2008 and December 2009 via adapted 4-point rating scales of positive/negative behaviors and affects. They found that counselors with Applied Suicide Intervention Skills Training (ASIST) were significantly more likely to positively impact caller behavioral and affect changes during the call, including callers feeling less depressed (OR 1.31; 95% CI 1.01–1.71; *p* < 0.05), less overwhelmed (OR 1.46; 95% CI 1.18–1.82; *p* < 0.05), less suicidal (OR 1.74; 95% CI 1.39–2.18; *p* < 0.001), and more hopeful (OR 1.35; 95% CI 1.04–1.77; *p* < 0.05), compared with counselors without ASIST (29). Furthermore, counselors with ASIST were significantly more likely to apply positive supportive and collaborative approaches, including exploring reasons for living (OR 1.46; 95% CI 1.03–2.07; *p* < 0.05) and ambivalence about dying (OR 1.65; 95% CI 1.19–2.28; *p* < 0.01). However, those with ASIST were not more likely to ask about suicide plans, preparatory behaviors/actions, intent, and prior suicide thoughts or attempts compared with counselors without ASIST (all *p* > 0.05) (29).

Gould and colleagues (32) (high risk of bias; Oxford quality rating of 4) cross-sectionally analyzed 491 calls to the NSPL between February and September 2012. This study is noteworthy because it consisted entirely of imminent risk callers and provided insights into the utilization of first responders to support crisis line services. Data were drawn from responder self-report questionnaires regarding imminent risk assessments and interventions provided. Interventions were classified according to four levels: active collaborative non-invasive; active collaborative invasive; active non-collaborative invasive; and, active non-collaborative noninvasive (See Supplementary Table 2). Collaborative calls included any active engagement by the caller to take action on her or his own behalf to work toward safety, and invasive interventions included the provision of emergency first responder services, sometimes referred to as “emergency rescues.” Results indicated that 76.4% of callers were collaborative in securing their own safety, and the remaining 24.6% required a non-collaborative and involuntary use of emergency services (32). A novel approach to developing risk profiles was also explicated by classifying callers along two continuums based on level of risk and level of engagement.

In the only study focused exclusively on crisis chat outcomes, Mokkenstorm et al. (35) (high risk of bias, Oxford quality rating of 4) analyzed 526 administrative records of chat logs from April to June 2013 to measure immediate proximal change from the beginning to end of a crisis chat, and found that suicidal ambivalence worsened for 15 of the users (2.9%), 156 (29.7%) had no change, and 18 improved (3.4%). Missing data was an issue for this outcome (337; 64.1%). The CCORS was also used to assess chatter's positive and negative experiences and behaviors. The mean score was 114.1 (Standard Deviation [SD] 16.8; range 61–150), though “a mixed picture emerges,” (p. 289) with 27.6% of chats rated to be dissatisfied, and 28.7% were satisfied; 33.1% said she or he did not seem to feel better, while 20.2% felt better (35).

In the most recently published study regarding immediate proximal evidence of effectiveness, Mejias-Martin et al. (40) (moderate risk of bias; Oxford quality rating of 4) analyzed 20,942 calls to the EPES public emergency healthcare service of Andalusia, Spain between January 2007 and December 2013. Based on records from the phone operator and healthcare team labeling, the researchers noted 516 caller deaths prior to evacuation (2.46% of analyzed calls), and that males died significantly more frequently than females (4 vs. 0.98%; *p* = 0.001) (40). Almost three-fourths (72.37%) of calls resulted in an emergency rescue evacuation to the emergency department, while 13.05% were resolved *in situ*, 4.61% were referred to a professional, and 1.96% denied to be attended (40). In analyses to understand groups with more frequent evacuation, callers over 65 years old had two times lower likelihood of evacuation compared with younger callers (adjusted OR 0.53; 95% CI 0.47–0.59), and females were more frequently evacuated compared with males (*p* = 0.001), while also having calls more frequently resolved *in situ* (40).

### Distal Evidence of Effectiveness

The remaining 17 studies measured more distal outcomes and were categorized by proximity of outcome measurement from the time of crisis line service. Distal evidence ranged from follow-up about 1 week after the crisis line service, to up to 4 years (See Supplementary Table 4). For three studies, the outcome measurement was distal but the time elapsed between the call and the follow-up was not clear. Distal evidence consisted largely of single-group before-after study designs measuring initial outcomes during the crisis line intervention, along with a single follow-up assessment after the crisis line service. Several before-after studies included multiple assessment timepoints for distal outcome measurement. One RCT measuring distal outcomes was noted, along with a few retrospective cohort studies. Crisis lines evaluated for distal outcomes also served both adult and adolescent populations, including one adolescent only study, five mixed adult/adolescent samples, seven adult only samples (including one US Veteran study), and four studies in which age was not reported. Distal studies were conducted in Canada (seven studies), the US (six studies), and one each from Amsterdam, China, Hungary, Belgium, and India. Distal outcomes measured included SDV (13 studies), client mood/satisfaction (eight studies), helper responses/approaches (four studies), the provision of referrals (six studies), as well as service utilization (seven studies).

Similar to the proximal studies, approaches to distal outcome measurement varied, including one study that utilized silent monitors, and four studies that incorporated validated assessment tools. However, five studies relied on administrative/clinical records, four studies used approaches not shown to be reliable or valid, and one study did not report source of data. Selected distal results are presented by proximity of the most distal outcome measurement to the crisis line service.

In the most proximal distal study with outcome measurement via follow-up calls at 1 week, Mishara and colleagues Study 2 (33) (high risk of bias; Oxford quality rating of 4) analyzed 1,206 calls to Quebec suicide prevention centers in Canada. Outcome measures included a mix of standardized and unstandardized approaches, including ratings by silent monitors, Helper Response Scales, CCORS, the Psychological Symptom Index (abridged), and the Brasington Indication of Depression. Significant decreases were noted in suicidal urgency from the beginning to the end of the call (*p* < 0.001), although there were no changes in 76% of calls. Additionally, suicidal urgency decreased in 16% of calls, but increased in 7.8% of calls (33). For this study, suicidal urgency was defined along a seven-point scale ranging from one (thinking about suicide with no plan, time frame, or method), to seven (decided to take own life in the next 24 h with a specific method determined and available). Follow-up outcomes regarding distal effectiveness of the crisis line were mixed and consisted of outcome data for just 8.7% of the baseline sample. At 1 week, 69.2% of callers were satisfied with help received, but 31% were not. A substantial proportion (42%) reported they did what they said they would do since the initial calls, but 40.2% admitted they did not (33). The authors also noted gender differences in effectiveness; female callers improved more frequently than males (18.6 vs. 11.8%; *p* < 0.05), and CCORS was significantly higher in females compared with males (*p* < 0.001) (33).

Kalafat et al. (18) (high risk of bias; Oxford quality rating of 4) analyzed 1,617 callers to local crisis hotlines and the 1-800-SUICIDE network from March 2003 to July 2004. Distal outcomes covering a variety of client domains were measured a mean 13 days from the baseline call (range 1–52 days) for about half (49.5%) of baseline callers. Assessment approaches were both standardized and unstandardized, including a 14-item measure adapted from the Profile of Mood States-A Modified (POMS-M) (47) and Likert scales. Findings revealed that 11.7% of callers had suicidal thoughts since the initial call (18). Callers who participated in the follow-up assessment were significantly more overwhelmed and received significantly more referrals compared with callers without follow-up (*p* < 0.001). POMS-M, caller distress, confusion, depression, anger, anxiety, helplessness, feelings of being overwhelmed, and hopelessness all significantly reduced from the beginning of the call to the end of the call and from the end of the call to the follow-up at 2 weeks (all *p* < 0.001) (18). 57.9% of those who completed follow-up initiated an action plan with their counselor, and among those only 35 had not carried out any of the plan. Among those who completed follow-up and had been referred to a mental health resource (392), 33.2% had kept or made the appointment at follow-up (18). Of the three rescues initiated during the crisis call, two completed follow-up and one did not.

Gould et al. (25) (high risk of bias; Oxford quality rating of 4) analyzed 654 NSPL callers between January 2006 and December 2007 who were referred to health care. Standardized telephone interviews were conducted a mean 14 days after the initial call to the center (range 3–72 days), and they included suicide risk status, Beck Depression Inventory-II (48), along with other unvalidated questions. Overall, 41.9% of callers followed through with their referral, with the highest follow-through rate to mental health providers (25). However, 151 suicidal callers did not follow through with a referral, albeit 25% of those reported accessing a comparable mental health resource. Utilizing a mental health referral was not related to demographics, depression, or suicidal risk profile, although unsurprisingly utilization rates were higher among those with insurance compared with those without insurance (59.6 vs. 35.6%; OR 0.37; 95% CI 0.19–0.72; *p* < 0.01), and among those already in treatment compared with those who were not (76.7 vs. 26.1%; OR 9.32; 95% CI 5,91–14.70; *p* < 0.0001) (25). Perceptions about barriers to utilizing mental health resources among crisis line callers were also explored.

In the only other RCT evaluating crisis line effectiveness (and the only RCT measuring distal effects), Mishara et al. (15) compared the effects of four suicide prevention program arms for crisis line callers between February 2000 and January 2002. This approach was unique in that the study participants were family and friends who had called the crisis line with concern about high-risk suicidal men who did not seek help themselves. Using a mixture of standardized and unstandardized assessment approaches, they found that overall, the crisis line caller participants reported that the suicidal men they were concerned about were significantly less likely to have seriously considered suicide after participation in any of the crisis line programs (at 2 months *p* < 0.001; at 6 months *p* < 0.01), and less frequently attempted suicide in the previous 2 months (at 2 months *p* < 0.02; at six months *p* < 0.001) (15). However, problems with the design and execution of this trial introduced high risks of bias and cast doubt about the validity of these findings. Issues included discrepancies in the reporting of number of participants, errors in the table reporting results, the abandonment of the family session arm of the trial due to lack of participation, and low completion and analysis rates with missing reasons for dropout.

In the most thorough examination of service utilization after a crisis call, Britton et al. (31) (high risk of bias; Oxford quality rating of 3) retrospectively investigated distal VCL effectiveness by examining caller service utilization within 180 days of index referral during the crisis call. Referrals were made for 21,130 callers (20.6% of all calls), and the analysis included 13,444 callers (64% of eligible referrals) during calendar year 2010. Based on precise linkage of VCL call records with VHA medical files, it was revealed that VCL is most frequently used by Veterans already engaged in VHA care (91% of the sample had prior VHA use within the past 5 years). The majority of callers presented for in-person VHA care within seven days of referral (71% of callers without prior VHA use and 91% with prior VHA use). Callers with prior VHA use were more likely to present for same-day care; however, callers without prior VHA use were more likely to present for care after 15 days (*p* < 0.0001) (31). There were few other differences in service utilization observed between the two groups.

The only study across the entire body of evidence rated as low risk of bias was by Chan et al. (38) who conducted a retrospective cohort study analyzing death by suicide among elderly users and non-users of a telephone helpline between January 2012 and December 2015 (Oxford quality rating of 3). Outcomes were assessed via sociodemographic data from the service's computerized system, as well as suicide mortality status from the Coroner's Court matched against crisis line users using the unique Hong Kong Identity Card. In this study, helpline users accounted for 14.4% of known suicides in Hong Kong during the 4 year follow-up period, and the suicide rate among helpline users was far higher than the general Hong Kong older adult population (Males: 86.3 vs. 32.6 per 100,000; Females: 42.8 vs. 16.7 per 100,000; both Incident Rate Ratio [IRR] = 2.6) (38). The majority (60%) of the helpline suicides occurred within 5 years of the service. Significant predictors of suicide among the helpline users included older age, male, living alone, and self-reported mental illness. Protective factors were also identified including skeletal system diseases and brain and nervous system diseases (38).

Work by Pil et al. (30) (moderate risk of bias; Oxford quality rating of 4) was unique in that the team modeled cost-effectiveness of Flemish suicide chat and phone helpline services using 2011 data from 3,785 users in a 10-year simulation to predict distal future effects. Findings suggested that telephone and chat crisis line services could avoid 36% of projected future suicide attempts and provide modest cost-savings.

### Volunteer vs. Paid Responders

Two studies provided additional insights into the effects of characteristics of crisis line responders on outcomes. These studies sought to identify differences between volunteer vs. paid responders (both high risk of bias; Oxford quality ratings of 4). In a study by Gould et al. (32) volunteers were significantly less likely to engage in a collaborative active rescue compared with non-volunteers (OR 0.41; 95% CI 0.23–0.74; *p* = 0.003), and volunteers were significantly more likely to implement a non-collaborative active rescue compared with non-volunteers (OR 2.31; 95% CI 1.40-3.81; *p* = 0.001) (see Supplementary Table 2). For each additional 4 h per week shift answering calls, helpers had 8% higher odds of collaboratively engaging caller (*p* = 0.006), 8% lower odds of implementing a non-collaborative rescue (*p* = 0.008), and 8% increased odds of reducing a caller's imminent risk so no rescue was needed (*p* = 0.03) (32). Mishara et al. (33) found that overall, there were no significant differences between volunteers and paid employees on outcomes. However, volunteers and paid staff with over 140 h of call experience had significantly better outcomes compared with those with less experience. More experienced helpers (140+ h) were less likely to have an increase in suicide risk from beginning to end of call (5.4 vs. 12.2%), more likely to have improvement in suicide urgency, defined along a seven-point scale from thinking about suicide with no plan to decide to take own life in the next 24 h with a specific method determined and available (16.8 vs. 14.7%; *p* < 0.02); significantly higher CCORS (46) scores (*p* < 0.025), and were more likely for the safety contract/agreement to be respected (50.1 vs. 31.1%; *p* < 0.04) compared with less experienced helpers (<140 h) (33).

## Discussion

### Summary of Main Findings

Although the state of the science regarding the effectiveness of crisis response services remains limited, overall results provide support for such services. However, such support is largely from uncontrolled studies indicating the positive effect of crisis line calls on immediate proximal outcome measures (e.g., changes in distress over the course of the crisis line call) and short-term distal effects. Many studies evaluating distal effects after the crisis service suffered from substantial dropout, thereby increasing the risk of bias interpreting findings. However, some distal studies utilizing administrative data were able to retain complete follow-up data [e.g., suicide mortality data (38); medical records (31)], but they did not benefit from participant self-report to contextualize findings. Cautious interpretation of Chan et al. findings is warranted. While the study found significantly higher rates of suicide among crisis line callers, this is not necessarily an indication of lack of crisis line effectiveness. Rather, this study confirmed that crisis line callers are at increased risk for suicide, reinforcing the need for high quality wrap-around services and follow-up care to promote recovery and well-being. While reliability of outcome measurement has been shown in some approaches (e.g., silent monitoring, rating transcripts), further research is needed to establish validity in outcome ascertainment (e.g., measure SDV using standardized assessment tools). Promising approaches to outcome measurement have incorporated validated assessment tools, often modified for brevity (e.g., CCORS, MINI, POMS, BDI); however, more research is needed.

### Strengths

The strengths of this review lie in the rigorous methodological approach utilized that is consistent with PRISMA guidelines. An in-depth examination of individual study characteristics combined with a descriptive synthesis of key features and findings contextualize the state of crisis line effectiveness research and illuminate opportunities for future studies. Strengths of the literature include an increased focus over the last decade on crisis line effectiveness evaluation research, in which almost two-thirds of included studies were published since 2010. The evidence is also growing to include research using longitudinal study designs with a comparison group [e.g., (38)], as well as a landmark RCT by Gould et al. (29) that used a dynamic wait-listed roll-out to evaluate a network of call centers. These exemplar studies prove that it is possible to implement rigorous and sophisticated study designs in the understandably complex and complicated field of crisis line evaluation. Current evidence supports the continuation and expansion of crisis line services as an important safety net for comprehensive suicide prevention care.

### Limitations

As outlined above, the limitations of the literature are that the overall quality of studies conducted to date are low, and risk of bias is concerning. Significantly less evidence was available to review in terms of crisis chat, and no studies have been conducted to evaluate the effectiveness of text-related services. In addition, there was substantial variability in what outcomes were measured, and the timing of those measurements. A key limitation emerged in defining what truly is effectiveness in crisis line evaluation. The measurement of effectiveness was discerned to be a multi-faceted domain covering much more than the central outcome to prevent suicide and other self-directed violence, and included measures of mood, satisfaction, referrals, and utilization/engagement in care. Furthermore, half of studies measured only immediate proximal outcomes of effectiveness, and studies measuring more distal outcomes widely varied in terms of time to follow-up for outcome measurement (1 week to up to 4 years). The inconsistent use of standardized tools to measure outcomes along with the variety of outcome domains made it challenging to integrate effects across studies, leading to uncertainty in the extent to which crisis line services truly are meeting their intended goals. Also notable are the high losses to follow-up as well as current dearth of evidence regarding the highest risk callers. That being said, such work is complicated by the imminent risk presented by such callers. Exploration of means to evaluate these interactions is warranted (e.g., reviewing recorded interactions).

Additionally, longer-term outcomes would be expected to be improved if crisis line users could be connected to behavioral health services. Most basically, this might include responders offering users resources regarding providers in their community. In particular, opportunities exist in terms of crisis lines following individuals until they engage in treatment. Though ultimately this is an empirical question, models exist, such as Safety Planning Intervention plus follow-up (SPI+) (49), that could be modified to meet the needs of crisis line service users. With that in mind, such interventions are contingent upon users being willing to self-disclose information regarding their identity. This runs counter to the historical anonymous culture of crisis services (50). This culture of anonymity poses clinical and research considerations in regards to challenges associated with providing users with follow-up care and evaluating distal effects of services. Moreover, such interventions are often dependent upon follow-up services being available. Progress on health equity in the US and other countries must remain a priority to meet the behavioral health follow-up service needs of crisis line users (51).

The limitations of this review are that included literature was limited to English language only, and synthesis was not quantitative (e.g., no meta-analysis was performed).

### Future Directions

Additional work is needed to evaluate the impact of responder experience on user outcomes. The most robust immediate proximal evidence from a national RCT indicates that counselors with ASIST had improved user outcomes during the call (29). Findings from both Gould et al. (32) and Mishara et al. (33) suggest that factors associated with responder characteristics impact outcomes. It remains unclear whether paid responders simply have more time to become “experienced.” It may also be that those who are paid receive additional resources (e.g., training) that support better outcomes. The evidence to date provides strong indications that responder experience improves outcomes, and it is imperative that all responders are trained to consistently incorporate standardized SDV risk assessment and develop a supportive/collaborative approach to assisting crisis line users. Future studies should incorporate participatory approaches to increase responder engagement in the research process. This will encourage the initiation of study procedures as part of a continuous feedback loop for quality improvement.

Computational linguistics and natural language processing are ripe evaluation paradigms to complement effectiveness research. Various linguistic aspects of conversations can be measured and correlated with crisis service outcomes. Natural language interfaces may be able to assist human responders in linguistic development (52) as well as provide real-time emotional and practical support to responders during crisis chat and text interactions (53, 54). It is critical that a rigorous framework of principles and protocols is applied to ensure the safe and ethical conduct of these research paradigms, as this approach requires the sharing of highly sensitive data between technology companies and crisis line academic researchers, as piloted in the Crisis Text Line platform (55).

### Conclusions

Despite the fact that research regarding the effectiveness of crisis line services remains limited, studies overall provide initial support for such services, particularly in terms of calls impacting immediate proximal and short-term distal outcomes. Crisis line callers are a high risk population, confirming the need for competent responders trained in suicide-specific assessment and care. Optimal models of crisis lines should implement proactive follow-up services that incorporate distal evaluation. Additional high quality research is needed particularly among the highest risk callers. Further exploration of proximal and distal outcomes regarding call, chat, and text services will benefit this population.

## Author's Note

A version of this work was previously presented: Hoffberg AS, Stearns-Yoder KA, Brenner LA (Oral presentation). The Effectiveness of Crisis Line Services: A Systematic Review of the Past 30 Years and Opportunities for the Future. 52nd American Association of Suicidology Conference. 2019 Apr 26; Denver, CO.

## Author Contributions

LB contributed conception and design of the study, and contributed to the selection of studies, grading of evidence, and interpretation of results. KS-Y contributed to the design of the study, protocol development, selection of studies, and grading of evidence. AH contributed to the design of the study protocol development, selection of studies, grading of evidence, interpretation of results, and wrote the first draft of the manuscript. LB and KS-Y wrote sections of the manuscript and contributed to manuscript revision. All authors have contributed substantially to the paper and read and approved the manuscript and its submission to this journal.

### Conflict of Interest

The authors declare that the research was conducted in the absence of any commercial or financial relationships that could be construed as a potential conflict of interest.
