# Supplementary material for: The Effectiveness of Crisis Line Services: A Systematic Review
Source: Front Public Health. 2020 Jan 17;7:399. doi: 10.3389/fpubh.2019.00399 (PMC6978712; doi:10.3389/fpubh.2019.00399)
Supplement: Supplementary file 1 [file Table_1.docx]

**Supplementary Table 1. OVID Medline Search Strategy**

1 exp Self-Injurious Behavior/

2 ((self adj3 (harm or injur* or violen* or destruct*)) or suicid* or selfharm or selfinjur* or parasuicid*).tw,kw.

3 1 or 2

4 exp hotlines/ or exp call centers/ or exp "cell phone use"/ or exp negotiating/

5 (hotline* or lifeline* or helpline*).tw,kw.

6 ((crisis or crises or critical or emergenc* or acute) adj5 (chat* or text* or call* or SMS or MMS or telephone or phone or line)).tw,kw.

7 4 or 5 or 6

8 3 and 7

9 limit 8 to english language

10 limit 9 to "animals"

11 limit 9 to "humans"

12 10 not 11

13 9 not 12
