# Supplementary material for: The Effectiveness of Crisis Line Services: A Systematic Review
Source: Front Public Health. 2020 Jan 17;7:399. doi: 10.3389/fpubh.2019.00399 (PMC6978712; doi:10.3389/fpubh.2019.00399)
Supplement: Supplementary file 2 [file Table_2.docx]

**Supplementary Table 2. Definitions**

| Term | Definition |
| --- | --- |
| Crisis phone line | A free, 24/7/365 telephone number people can call to get immediate, anonymous, and confidential crisis intervention and emotional support by trained specialists. |
| Crisis text line | A free, 24/7/365 SMS (short message service) number people can text to get immediate, anonymous, and confidential crisis intervention and emotional support by trained specialists via a secure online platform. |
| Crisis chat line | A free, 24/7/365 online chat service people can message through an internet browser to get immediate, anonymous, and confidential crisis intervention and emotional support by trained specialists via a secure online platform. |
| Effectiveness | Assessment of the immediate proximal and/or longer-term distal effect(s) of the crisis line service intervention on all health- and service use-related client outcomes regarding self-directed violence, mood, satisfaction, compliance, and/or service utilization, as well as responder responses such as intervention style and referral recommendations. |
| Proximal outcomes | Short-term outcomes measured during and/or immediately following a crisis line service. |
| Distal outcomes | Longer-term outcomes measured after the end of a crisis line service. |
| Silent monitor | A methodology to measure crisis line effect in which trained investigators unobtrusively listen to calls and rate the nature of help provided, characteristics of responders, caller reactions, and observed impact on callers, often using standardized rating scales. In some cases, interrater reliability is established via independent simultaneous observation of calls. |
| Active collaborative noninvasive | Callers engaging with responders to take action on own behalf and work towards securing their own safety without the provision of emergency services (e.g., voluntarily involving 3^rd^ party, transporting self to additional care) |
| Active collaborative invasive rescue | Callers engaging with responders to take action on own behalf and work towards securing their own safety with the provision of emergency services "rescues" |
| Active noncollaborative noninvasive | Involuntary interventions without caller consent to secure caller safety without the provision of emergency services (e.g., involving 3^rd^ party, sending outreach team) |
| Active noncollaborative invasive | Involuntary interventions without caller consent to secure caller safety with the provision of emergency services "rescues" |
| Emergency rescue | Emergency procedure in which local emergency services are contacted by crisis line responders when a caller has initiated a suicide attempt and his or her life appears to be in imminent danger |
