# Supplementary material for: The Effectiveness of Crisis Line Services: A Systematic Review
Source: Front Public Health. 2020 Jan 17;7:399. doi: 10.3389/fpubh.2019.00399 (PMC6978712; doi:10.3389/fpubh.2019.00399)
Supplement: Supplementary file 4 [file Table_4.docx]

# Supplementary Table 4. Proximal and Distal Evidence on Crisis Line Effectiveness

| **Article** | **Country** | **Crisis Line Description** | **Sample Description (age, gender, suicidal thoughts/behavior)** | **Study Time Period** | **Effectiveness Domain(s)** | **Source(s) of Outcome Data** | **Proximity of Outcome Measurement** | **Findings** |
| --- | --- | --- | --- | --- | --- | --- | --- | --- |
| Table 4a. Proximal Evidence on Effectiveness | | | | | | | |  |
| de Anda & Smith,[42] 1993 | USA | 2 suicide help lines in Los Angeles County, n=1 operated by major suicide prevention center, n=1 peer crisis helpline for adolescents | n=405 callers analyzed  Age & Gender, n=165 12-19 years; n=116 females (70.3%); n=65 20-26 years; n=35 females; n=175 27+ years; n=102 females  Suicidal thoughts/ behavior, NR | 2-week period (dates not reported) | Helper response | Call sheet routinely used by volunteers in the agency; Additional brief checklist at 2^nd^ agency | During call | **Helper response:**  n=54 adolescents referred for services (32.7%); n=15 to mental health/ counseling (9.1%); n=5 to sex information (3.0%); n=8 to family planning (4.8%); n=1 to Los Angeles suicide prevention center (0.6%); n=3 to child abuse program (1.8%); n=3 to drug abuse program (1.8%); n=2 to alcohol program (1.2%); n=1 to gay/ lesbian program (0.6%); n=3 to family & friends (1.8%); n=1 to emergency medical treatment (0.6%); n=8 to other community agency (4.8%); n=1 traced call (0.6%); n=1 to gang hot line (0.6%)  n=29 young adults referred for services (44.6%); n=19 to mental health/ counseling (29.2%); n=10 to other community agency (15.4%)  n=77 adults referred for services (44.1%); n=47 to mental health/ counseling (26.9%); n=29 to other community agency (16.6%); n=1 traced call (0.6%) |
| King et al.,[21] 2003 | Australia | Kids Help Line | n=100* taped calls analyzed (5.5% of all suicide-related calls)  Age, NR  Gender, n=58 female (79.5% of identifiable)  Suicidal thoughts/ behavior, 100% suicide- related; n=93 (92.1%*) clear suicidal ideation; n=8 (7.9%) general thought better off dead or intent to self-harm without wish to die  *Discrepancies in report, n=101 callers; 92.5% with suicidal ideation | March 1998 – March 1999 | Caller suicidal thoughts/ behaviors  Caller mood  Helper response | Two independent raters of caller suicidal ideation, intent, & mental state using items adopted from MINI Modules A & C  6 items rating mental health on 3-point scale (anger/ irritability; sadness/ tearfulness; negativity/ hopelessness; distress/ agitation; guilt/ shame; slowed/ nonspontaneous speech)  5 items rating suicidal ideation using dichotomous scale  4 items rating suicidal urgency using dichotomous scale that were recoded into 5-item ordinal scale | Change from beginning to end of call via rating 10 minutes of each tape, 5 minutes from time suicidal ideation first evident & final 5 minutes of interview | **Caller suicidal thoughts/ behaviors:**  significant decrease in suicidal ideation (p<0.0005)  substantial decrease in proportion of cases rated imminent risk by both raters from beginning to end of call (47.5% vs 7%); substantial increase in proportion of callers rated as no suicide urgency risk from beginning to end of call (2% vs 58.5%) (p<0.001)  14% of callers remained suicidal at end of call  **Caller mood:**  significant improvement in mental state (p<0.0005)  **Helper response:**  n=14 3-way urgent referrals initiated during call |
| Latzer & Gilat,[43] 2006 | Israel | ERAN national telephone helpline run by all volunteers | n=147 calls where eating disorder identified by volunteer as main problem analyzed (0.4%)  Gender, n=133 female (90%)  Age, n=31 11-18 years (21%); n=34 19-25 years (23%); n=82 30-69 years (56%)  Suicidal thoughts/ behavior, n=7 suicidal callers (5%) | 1998 | Helper response | Standard report forms routinely completed in all ERAN centers | During call | **Helper response:**  56% ED-NOS, 30% AN, 30% BN, & 6% BED referred to treatment; significant difference between groups (p<0.01) |
| Mishara et al.,[23, 24] 2007 | USA | Hopeline Network (1-800-SUICIDE), a central toll-free number that transfers calls from anywhere in US to an accredited participating helpline center (n=91) | n=1,431 adult crisis calls analyzed (54.8% of all calls) from n=782 crisis helpers at n=14 centers; n=4 centers directive model; n=4 centers nondirective model  Gender, female to male ratio about 3:2  Age, 47% 18-34 years, 44% 35-54 years, 6% 55-65 years, 1% 65+ years  Suicidal thoughts/ behavior, n=503 suicide crisis calls (35.15%); n=33 attempt in progress (2.3%); n=182 attempt & plan (12.7%); n=288 expressed intent (20.1%) | August 19, 2003 – May 31, 2004 | Caller mood  Helper response | 11 within-subject ratings by 2 silent monitors on dimensional mood/ state of caller  CCORS  4 factors of helpers’ behaviors/ styles: supportive approach & good contact, active listening, collaborative problem solving, & negative approach; helper’s empathy, respect, & directivity  Suicide risk assessment | Change from beginning to end of call[23]  During call[23, 24] | **Caller mood:**  overall significant positive mean effect (p<0.001); apprehensive/ confident, n=160 decreased (11.2%), n=705 no change (49.2%); n=548 improved (38.3%); sad/ happy, n=131 decreased (9.1%), n=965 no change (67.4%), n=318 improved (22.2%); tired/ dynamic  n=211 decreased (14.7%), n=961 no change (67.1%), n=241 improved (16.8%); helpless/ resourceful, n=136 decreased (9.5%), n=581 no change (40.6%), n=698 improved (48.7%); hopeless/ hopeful, n=159 decreased (11.1%), n=677 no change (47.3%),n=575 improved (40.2%); confused/ decided, n=142 decreased (9.9%), n=520 no change (36.3%),n=749 improved (52.3%); crying, n=52 decreased (3.6%), n=1,207 no change (84.3%), n=154 improved (n=10.8%); depressive mood, n=94 decreased (6.6%), n=1,058 no change (73.9%), n=262 improved (18.3%); desperate, n=89 decreased (6.3%), n=1,098 no change (76.7%), n=225 improved (15.7%); agitated, n=66 decreased (4.6%), n=1190 no change (83.1%), n=155 improved (10.8%); ambivalent, n=24 decreased (1.7%), n=1,006 no change (70.3%), n=165 improved (11.5%)  CCORS: M=102.4 (SD=18.51), significant differences between centers (p<0.03)  **Helper response:**  supportive approach & good contact associated with positive mood/ state changes (p<0.001); active listening associated with more crying (p<0.008); collaborative problem solving associated with positive mood/ state changes (p<0.001); negative approach not significant; supportive approach & good contact, problem solving related to higher CCORS scores (r^2^=0.10); all 4 styles associated with reaching agreement for no-harm contract (p not reported), negative style negatively related; higher empathy & respect were significantly associated with higher CCORS scores (p<0.001), fewer hang ups (p<0.001), reaching a no-contract agreement (p not reported), & mood/ state improvements (p<0.001); mixed directivity associated with highest CCORS scores & mood/ state changes, followed by high directivity (p<0.001), no relationship between directivity & caller hang ups or reaching no-contract agreement; n=223 calls helper failed to meet minimum acceptability (15.6%), n=87 lack of empathy, n=31 lack of respect, n=73 poor initial contact; n=4 encouraged suicide; n=87 helper expressed unable to help; n=46 helper interrupted call; n=76 helper was aggressive or rude; n=37 helper did not discuss problem with caller.  n=723 not asked about SI (50.5%); among n=474 with SI, n=219 not asked about plan (46.2%); among n=159 with plan, n=119 not asked if attempt in progress (74.8%); among n=33 with attempt in progress, n=6 dispatched emergency services (18.1%), helper stayed on the line until arrival in n=3 cases (50%); among n=18 remaining attempts, n=8 changed their mind/stopped the attempt (44.4%), n=4 hang ups (22.2%), n=3 call ended mid-attempt (16.6%), n=1 helper hung up after caller became unconscious(5.5%); n=2 emergency rescues initiated by research team  among n=503 suicidal callers, supportive approach & good contact, collaborative problem solving associated with higher CCORS scores (r2=0.14); among n=215 high-risk suicidal callers, only supportive approach & good contact associated with higher CCORS scores (r^2^=0.13); only supportive approach & good contact associated with reaching no-harm contract with suicidal (OR=1.78; 95% CI=1.481-1.993) & high-risk suicidal (OR 1.76; 95% CI=1.397-2.221) callers; among suicidal callers, supportive approach & good contact, & collaborative problem solving associated with fewer caller hangs ups (OR =0.558; 95% CI 0.444-0.702; OR=0.794; CI 0.640-0.985, respectively), only supportive approach & good contact associated with no-harm contracts among high-risk suicidal callers (OR not reported) |
| Coveney et al.,[44] 2012 | UK | Samaritan crisis line, an anonymous volunteer line | n=1,309 survey responses analyzed (93.8% of completed)  Gender, n=1,002 female (77.9%)  Age, n=131 <16 years (10.1%), n=470 16-24 years (36.1%), n=253 25-35 years (19.4%), n=188 36-44 years (14.5%), n=181 45-54 years (13.9%), n=67 55-64 years (5.1%), n=8 65-74 years (0.6%), n=1 75-84 years (0.1%), n=2 85+ years (0.2%); n=8 missing (0.6%)  Suicidal thoughts/ behavior, n=606 felt suicidal (46.3%); n=113 in process of suicide (8.6%) | May 2008 – May 2009 | Caller mood  Caller satisfaction  Helper response | Online survey on use of, experience, & assessment of service  How user felt at end of last contact on 10-point semantic differential scale (unhappy/ happy; not listened to/ listened to; alone/ not alone; afraid/ unafraid; anxious/ not anxious; unhappy/ happy; not confident/ confident; not understood/ understood; hopeless/ hopeful; depressed/ not depressed; don’t want to live/ want to live; not cared for/ cared for; not supported/ supported; no solution/ solution found; no advice/ advice given) | After end of call | **Caller mood/ satisfaction:**  MS=7 overall feeling (IQR 5-8); MS=6 felt better at survey compared with immediately after contact (IQR 3-8); MS=9 listened to (IQR 7-10); MS=7 suicidal (IQR 4-10); MS=6 lonely (IQR 4-8); MS=6 afraid (IQR 4-8); MS=6 anxious (IQR 4-8); MS=5 happy (IQR 2-6); MS=5 confident (IQR 3-6.25); MS=7 understood (IQR 4-9); MS=6 hopeful (IQR 3-7); MS=4 depressed (IQR 2-7); MS=6 wanted to live (IQR 3-9); MS=5 cared for (IQR 2-6); MS=6 supported (IQR 3-9); MS=7 solution found (IQR 5-9); MS=8 given advice (IQR 5-10); MS=7 helpfulness of last contact (IQR 5-9); MS=8 overall perception of helpfulness (IQR 7-10); M=8 perception of service compared with expectations (IQR 5-9); n=673 felt always listened to (62%); n=329 felt sometimes listened to (30.4%); n=33 did not feel listened to very often (3.1%); n=46 did not feel listened to at all (4.3%); n=357 reported consistent level of service across contacts (66%); n=268 rated service excellent (31.8%); n=332 rated service good (39.4%); n=123 rated service reasonable (14.6%); n=40 rated  service bad (4.8%); n=79 rated service variable (9.3%); n=408 provided suggestions for improvement (40%); n=425 in contact with other services (38%); n=278 statutory services (84.2%); n=72 statutory more helpful (26.1%); n=101 equal (36.6%); n=103 statutory less helpful (37.3%); n=89 voluntary services (27%); n=25 voluntary more helpful (29.8%); n=31 equal (36.9%); n=28 voluntary less helpful (33.3%); n=26 alternative services (7.9%); n=9 alternative more helpful (34.6%); n=12 equal (42.2%); n=5 alternative less helpful (19.2%)  **Helper response:**  n=655 reported being asked whether feeling suicidal at last contact (59%) |
| Knox et al.,[45] 2012 | USA | VCL | n=171,000 calls analyzed  Gender, 30% female  Age, 40-69 years  Suicidal thoughts/ behavior, NR | July 2007 – September 2010 | Helper response | Referrals to SPC & other VA/ community programs (source NR) | During call | **Helper response:**  approximately 4,000 referrals were made to SPCs as of 2008; n=16,000 SPC referrals at the end of September 2010; in addition to these referrals, simultaneous referrals were made to diverse programs in the VA, including programs for returning Veterans  from the wars in Afghanistan & Iraq, programs for women, programs for homeless Veterans, & substance abuse services; community referrals were made  for Veterans not eligible for care within the VA (n’s not reported) |
| Tan et al.,[46] 2012 | Canada | Nunavut Kamatsiaqtut Help Line | n=2,858 distress calls analyzed (71.92% of all calls)  Gender, n=1,674 females (54.36%)  Age, n=1,497 adults (81.80%); n=251 youth (14.89%)  Suicidal thoughts/ behavior, n=284 callers with suicidal thoughts or intentions (8.05%) | 1991 - 2001 | Caller satisfaction  Helper response | Type of assistance provided by crisis line volunteers via anonymized call logs analyzed by independent raters | During call | **Caller satisfaction:**  several callers noted benefits of crisis line, including feeling better, release of emotional tension in confidential environment, gaining clearer perspective, & arriving at potential solutions; many expressed gratitude & blessings to volunteers, sometimes calling for that specific purpose  **Helper response:**  n=3,662 codes for types of assistance; n=1,488 empathetic listening responses (40.63%); n=1,141 referral recommendation responses (30.07%); n=760 offers of suggestion (20.75%); n=273 advised caller to call back because volunteer not available (4.55%); referral sources mentioned included social services (32.81%), medical services (18.07%), clergy (11.67%), drug & alcohol treatment (8.86%), law enforcement (8.77%), shelter homes (3.68%), psychiatric/ psychological services (3.07%), legal services (2.81%), & elder (1.40%); females more likely to receive suggestions on how to resolve problems (n=498 vs n=259; p<0.001), directed to law enforcement service (n=68 vs n=32; p<0.05), shelter homes (n=34 vs n=8; p<0.001), social services (n=250 vs n=123; p<0.001), medical services (n=147 vs n=56; p<0.001), & other resources (n=63 vs n=36; p<0.01) compared with males; males more likely to be referred to psychiatric/ psychological services compared with females (p<0.001); helpers more likely to respond with empathetic listening & suggestions with adult callers compared with youth callers (p<0.05) |
| Britton et al.,[28] 2013 | USA | VCL | n=646 calls with complete data analyzed (40% of all calls)  Gender, n=100 female (16%)  Age, NR  Suicidal thoughts/ behavior, 100% Veterans with recent suicidal ideation or history of attempted suicide; n=217 categorized as higher risk (34%); n=429 categorized as lower risk (66%) | October 1-7, 2010 | Helper response | Responders action(s) at end of call: 1) referral sent to SPC; 2) caller declined referral but accepted SPC contact information; 3) caller declined referral & SPC contact information; 4) caller referred to local Veteran Service Center; 5) for California callers, warm transfer to health care provider; 6) caller was not suicidal at end of call (resolved); 7) caller accepted local number to obtain further assistance; 8) no action possible; classified as: resolved (6); referred (1, 2, 4, 5, 7); unresolved, declined referral (3, 8) | End of call | **Helper response:**  84% of calls ended with favorable outcome (n=162, 25% with resolution & n=380, 59% with referral to local health care provider); n=104 (16%) unresolved/ unreferred; 41% of calls did not lead to referral (23% higher risk, 51% lower risk); high-risk callers had greater odds of ending in referral (77% vs 49%; RRR 2.70; 95% CI 1.64-4.47, univariate), as did weekday calls during the day; callers at higher risk compared with lower risk approached significance in more calls resolved compared with unresolved/ declined referral (RRR 0.56; 95% CI 0.30-1.04; p=0.067, multivariate); responders used caller reported intent to die (OR 8.47; 95% CI 3.85-18.63) & absence of future plans (OR 10.45; 95% CI 2.84-38.40) to determine caller risk (higher vs lower); these optional data were available for 54% of calls |
| Gould et al.,[15] 2013 | USA | NSPL network of crisis hotlines | n=1,507 calls analyzed (54.3% of eligible calls) from n=1,410 individuals to n=17 centers; n=646 calls without ASIST training; n=764 calls with ASIST training  Gender, NR  Age, NR  Suicidal thoughts/ behavior, 100% acknowledged current thoughts of suicide, plans to kill self, or had taken action to kill self within day of calling | June 2008 – December 2009 | Caller mood  Helper response | Silent monitors blind to center ASIST training status assessed changes in counselor behavior via adapted 4-point scales of positive/ negative behaviors; overall assessment of effectiveness on single item 5-point scale; applying ASIST components to intervention (connecting, understanding, & assisting); changes in caller behavior via 7 items on 4-point scale based on caller affect & statements made during call (agitated, alone, depressed, overwhelmed, suicidal, confidence/ control, hope) | During call | **Caller mood:**  counselors with ASIST training were significantly more likely to have callers feeling less depressed (OR 1.31; 95% CI 1.01-1.71; p<0.05), overwhelmed (OR 1.46; 95% CI 1.18-1.82; p<0.05), suicidal (OR 1.74; 95% CI 1.39-2.18; p<0.001), & more hopeful (OR 1.35; 95% CI 1.04- 1.77; p<0.05) during the call, compared with counselors without ASIST training; each of counselor interventions that ASIST training impacted was significantly associated with positive caller behavioral changes  **Helper response:**  counselors with ASIST training had significantly longer calls (p<0.02) & increased number of invitations (connecting) (p<0.0001); significantly more likely to link invitations to suicidal thoughts (understanding) (OR 2.10; 95% CI 1.49-2.95; p<0.0001); explore reasons for living (OR 1.46; 95% CI 1.03-2.07; p<0.05); explore ambivalence about dying (OR 1.65; 95% CI 1.19-2.28; p<0.01); explore informal support contacts as part of safe plans (assisting) (OR 1.50; 95% CI 1.11-2.04; p<0.01) compared with counselors without ASIST training; counselors with ASIST training were no more likely to ask about or explore suicide plans, preparatory behaviors/ actions, intent, prior suicide thoughts or attempts, have more positive behaviors, or more overall effective, compared with counselors without ASIST training (all p>0.05) |
| Gould et al.,[29] 2016 | USA | NSPL network of crisis hotlines | n=491 call reports analyzed from n=132 helpers at n=8 crisis centers  Gender, 51.1% female  Age, NR  Suicidal thoughts/ behavior, 100% individuals identified by helper at imminent risk of suicidal behavior | February – September 2012 | Helper response | Helper self-report questionnaire on imminent risk & interventions provided (active collaborative invasive / noninvasive, active noncollaborative invasive/ noninvasive) | During call | **Helper response:**  n=375 callers with any active engagement (collaborative) (76.4%); n=344 with less invasive intervention (68.0%); n=94 with more invasive intervention (emergency services) (19.1%); n=35 calls involved both active rescue & active engagement; n=136 callers actively rescued (noncollaborative) (27.7%); n=18 less invasive intervention (3.7%); n=121 more invasive intervention (emergency services) (24.6%); n=192 imminent risk reduced enough so no rescue needed (39.1%); n=188 less invasive intervention (97.9%); n=9 caller transported self or 3^rd^ party transported to hospital; n=2 3^rd^ party involved without caller consent (1.0%); n=4 no interventions endorsed by helper (2.1%); n=299 imminent risk was not reduced by end of call & active rescue needed (60.9%); n=213 emergency services sent (71.2%); n=94 rescued collaboratively (44.1%); n=121 rescued noncollaboratively (56.8%); n=2 both collaborative & noncollaborative emergency rescue; n=86 less invasive intervention (28.8%); n=67 collaborative less invasive; n=26 caller agreed to transport self/ 3rd party transport to hospital; n=13 noncollaborative less invasive, such as involving VHA or mobile crisis team; n=11 no interventions endorsed by helper (3.7%); n=99 calls attempt in progress; n=76 emergency service sent (76.8%); n=47 emergency service sent noncollaboratively (47.5%); n=30 emergency service sent collaboratively (30.3%); n=1 both; n=10 calls imminent risk reduced, no emergency service needed (10.1%); n=11 less invasive intervention, not emergency services (11.1%); n=2 no interventions endorsed by helper (2.0%); volunteers were significantly less likely to engage in a collaborative active rescue compared with non-volunteers (OR 0.41; 95% CI 0.23-0.74; p=0.003); volunteers were significantly more likely to implement a noncollaborative active rescue compared with non-volunteers (OR 2.31; 95% CI 1.40-3.81; p=0.001); for each additional 4 hours/ week shift answering calls, helper had 8% higher odds of collaboratively engaging caller (p=0.006), 8% lower odds implementing noncollaborative rescue (p=0.008), & 8% increased odds reducing caller imminent risk so no rescue needed (p=0.03); latent classes: class I (high risk, moderate-high engagement), nearly ½ of sample, high scores on suicidal desire, over ½ engaged in preparatory behaviors or attempt in progress, low scores on buffers, over 2/3 high engagement with helper; class II (lowest risk, highest engagement), 21.4% of sample, highest proportion of low scores on suicidal desire, preparatory behavior present on over ¼ calls, rarely attempt in progress, high rates of buffers, near universal high engagement with helper; class III (moderate-high risk, lower engagement), 18.3% of sample, high/don’t know scores on suicidal desire, about ¼ attempt in progress, few with high reasons for living, majority high engagement with helper; class IV (highest risk, lowest engagement), 11% of calls, many unknown risk/protective factors, 75%  unknown suicidal desire, almost all high intent to die, highest rate of attempts in progress, lowest engagement with helper; class II significantly more likely to engage with helper, significantly less likely to implement active rescue, & significantly more likely to have reduction in imminent risk so no rescue needed compared with other classes; class I had significantly greater odds of imminent risk reduced so no rescue needed compared with classes III & IV; active engagement significantly associated with attempt in progress (OR 0.29; 95% CI 0.16-0.52), current intoxication (OR 0.48; 95% CI 0.27-0.86); & sense of purpose (OR 5.68; 95% CI 1.90-16.95); active rescue significantly associated with attempt in progress (OR 3.39; 95% CI 2.00-5.71) & sense of purpose (OR 0.19; 95% CI 0.07-0.48); imminent risk reduced so no rescue needed significantly associated with reasons for dying (OR 0.32; 95% CI 0.13-0.60), attempt in progress (OR 0.12; 95% CI 0.05-0.29), current intoxication (OR 0.52; 95% CI 0.29-0.94), & sense of purpose (OR 5.35; 95% CI 2.58-10.99) |
| Tyson et al.,[47] 2016 | UK | Rethink Mental Illness telephone helpline in Gloucestershire | n=87 callers agreed to participate & analyzed (15% of all callers)  Gender, NR  Age, NR  Suicidal thoughts/ behavior, n=59 felt the need to self-harm (68%); n=46 experienced suicidal thoughts (53%) | 1-year period (dates not reported) | Caller suicidal thoughts/ behaviors  Caller mood | Callers asked about mental state at beginning & end of call with 5-item Suicide & Self-Harm Evaluation Scale developed by research team | Change from beginning to end of call | **Caller suicidal thoughts/ behaviors:**  self-harm subgroup, n=59 callers felt the need to self-harm at beginning of call (68%) compared with n=24 at end of call (28%); 25% reduction in callers stating there was a significant or definite change of acting on self-harming thoughts; callers decreased in likelihood of acting on self-harming thoughts at end of call compared with beginning (M=3.4 vs M=2.3; p=0.00); suicidal ideation subgroup, n=46 callers reported experiencing suicidal thoughts at beginning of call (53%) compared with n=31 at end of call (36%); callers decreased in likelihood of acting on suicidal thoughts at end of call compared with beginning (M=3.2 vs M=2.6; p=0.00); n=1 caller stated definitely likely to act on suicidal thoughts at end of call compared with n=0 at beginning of call; n=12 first-time callers reported significant reductions in self-harm intent & suicidal ideation at end of call compared with beginning (p<0.05 on all questions)  **Caller mood:**  self-harm subgroup had significantly better mood at end of call compared with beginning (M=1.9 vs M=2.8; p=0.00); significant or definite change of feeling better tomorrow at end of call compared with beginning (M=2.01 vs M=2.5; p=0.00); suicidal thoughts subgroup had significantly better mood at end of call compared with beginning (M=1.5 vs M=2.0; p=0.001); increased confidence improved mood would be sustained tomorrow at end of call compared with beginning (M=1.2 vs M=1.5; p=0.007) |
| Mokkenstorm et al.,[13] 2017 | Amsterdam | 113Online volunteer-operated crisis chat service | n=526 crisis chat logs analyzed (30.4% of all logs) with n=78 helpers  Gender, n=382 female (72.6%)  Age, 22.6% <18  years; 53.6% 18-34 years; 17.7% 35-54 years; 1.7% 55+ years; n=25 age unknown  Suicidal thoughts/ behavior, n=451 suicide crisis (86.1%); n=20 attempt in progress (3.8%); n=111 had attempt plan (21.2%); n=320 expressed intent (61.1%); n=73 nonsuicide crisis (13.9%) | April 1 – June 1, 2013 | Chatter suicidal ambivalence  Chatter mood  Chatter satisfaction | Noncompulsory prechat questionnaire  CCORS  visitor’s emotional states coded by 2 independent raters (5-point Likert scale apprehensive/ confident; sad/ happy; helpless/ resourceful; tired/ dynamic; hopeless/ hopeful; confused/ decided); suicidal ambivalence (3-point Likert scale wants to die/ ambivalent/wants to live) | Change from beginning to end of chat as measured by first & last 10 minutes of logs | **Chatter suicidal ambivalence:**  n=15 deteriorated (2.9%); n=156 no change (29.7%); n=18 improved (3.4%); n=337 missing data (64.1%)  **Chatter mood/ satisfaction:**  CCORS, M=114.1 (SD 16.8; range 61-150); 27.6% visitors rated to be somewhat to very dissatisfied; 28.7% visitors rated to be somewhat to very satisfied; 33.1% visitors did not seem to feel better; 20.2% felt somewhat to a lot better; 46.3% of chats visitor mentioned new or more effective forms of coping during chat; chatter emotional states, apprehensive/ confident, n=14 deteriorated (2.7%); n=254 no change (48.3%); n=257 improved (48.9%); sad/ happy, n=2 deteriorated (0.4%); n=282 no change (53.6%); n=241 improved (45.8%); tired/ dynamic, n=25 deteriorated (4.8%); n=465 no change (88.4%); n=29 improved (5.5%); helpless/ resourceful, n=65 deteriorated (12.4%); n=255 no change (48.7%); n=197 improved (37.5%); hopeless/ hopeful, n=18 deteriorated (3.4%); n=252 no change (47.9%); n=255 improved (48.5%); confused/ decided, n=70 deteriorated (13.4%); n=227 no change (43.2%); n=221 improved (42.0%); crying, n=8 deteriorated (1.5%); n=498 no change (94.7%); n=18 improved (3.4%); depressive mood, n=5 deteriorated (1.0%); n=328 no change (62.4%); n=190 improved (36.1%); desperate, n=14 deteriorated (2.7%); n=281 no change (53.4%); n=229 improved (43.5%) |
| Ramchand et al.,[26] 2017 | USA | Suicide prevention hotlines in California | n=241 calls monitored (88.3% of screened calls) from n=10 accredited hotlines; n=6 NSPL centers  Gender, 57% females overall (range 30-77%)  Age, NR  Suicidal thoughts/ behavior, 21% thinking of suicide | Spring/ Summer 2014 | Caller mood  Caller satisfaction  Helper response | Call monitors ratings of overall call quality via single item 3-point scale  caller distress via single item 4-point scale  caller satisfaction via single item 5-point scale  suicide risk assessment according to adherence to NSPL- recommended standards  responses to callers via 12 ratings (7  positive & 5 negative) on a 3- point scale  referrals provided | Change from beginning to end of call | **Caller mood:**  M=43% decreased caller distress (range 28-64%), n=1 center significant reductions in distress; M=30% no change (range 11-54%); M=0% increased distress (range 0-15%); decreased distress associated with longer calls (p=0.029), member of lifeline (OR 2.72; p=0.024)  **Caller satisfaction:**  M=3.4 (range 3.2-3.9); higher satisfaction associated with longer calls (p<0.001), less challenging caller (p=0.001); callers who brought up interpersonal problems (p=0.017); having crisis chat & text (p=0.014)  **Helper response:**  overall call quality, M=66% good contact (range 43-88%); M=24% good contact with some weaknesses (range 9-46%); M=7% good contact not established/ important weaknesses (range 0-17%); suicide risk assessment, 69% asked about current ideation (range 13-100%); 25% asked about recent ideation (range 4-77%); 21% asked about past suicide attempt (range 0-60%); NSPL centers were more likely to ask about current ideation (77% vs 52%; OR 3.6; p<0.01), recent ideation (31% vs 16%; OR 2.5; p=0.02); & past attempts (27% vs 10%; OR 3.7; p<0.01); positive responses, M=1.8 allowed caller to talk about feelings/ situation (range 1.5-2.0); M=1.0 reflected back caller feelings (range 0.5-1.5); M=1.2 reflected back caller situation (range 0.8-1.6); M=1.5 connected/ established rapport with caller (range 1.2-1.8); M=1.6 sensitive/ receptive to caller problems (range 1.2-1.8); M=1.8 respectful to caller (range 1.3-2.0); M=1.0 showed empathy/ validated caller (range 0.5-1.4); negative responses, M=0 challenged caller in negative way (range 0-0.1); M=0 condescending to caller (range 0); M=0 displayed inappropriate behavior to caller (range 0-0.1); M=0 judgmental (range 0-0.2); M=0 preached to caller (range 0-0.2); referrals, 30% of all calls required referral; M=15% referrals provided (range 0-46%); M=15% referrals provided with some weaknesses (range 0-46%); n=5 centers never put callers on hold; M=7% of all calls put on hold (range 0- 26%) |
| Rasmussen et al.,[48] 2017 | USA | VCL | n=3,608 calls from Veterans who consented to SPC referral analyzed (98.6% of eligible calls)  Gender, 6.44% female  Age, n=2,707 calls from Veterans <60 years (75.03%); n=901 calls from Veterans 60+ years (range 60-101) (24.97%)  Suicidal thoughts/ behavior, 28.97% reported thoughts of suicide | July 2013 – December 2013 | Caller mood | Real-time clinical data obtained as standard practice during each call | End of call | **Caller mood:**  83.13% older callers & 82.56% younger callers reported feeling better at end of call |
| Mejias-Martin et al.,[30] 2018 | Spain | EPES public emergency healthcare service of Andalusia | n=20,942 calls analyzed (0.31% of all calls)  Gender, n=10,676 female (50.98%)  Age, n=772 15-19 years (3.69%); n=1,261 20-24 years (6.60%); n=1,685 25-29 years (8.82%); n=2,200 30-34 years (11.51%); n=2,533 35-39 years (13.26%); n=2,735 40-44 years (14.31%); n=2,260 45-49 years (11.83%); n=1,817 50-54 years (9.51%); n=1,032 55-59 years (5.54%); n=711 60-64 years (3.72%); n=562 65-69 years (2.94%); n=457 70-74 years (2.39%); n=385 75-79 years (2.01%); n=403 80-84 years (2.11%); n=296 >85 years (1.55%)  Suicidal thoughts/ behavior, 100% calls related to suicide attempts | January 1, 2007 – December 31, 2013 | Caller death  Helper response | Phone operator or healthcare team labeling | During/ after end of call | **Caller death:**  n=516 deaths prior to evacuation (2.46%); males more frequently died compared with females (4% vs 0.98%, p=0.001)  **Helper response:**  n=875 no action taken (4.18%); n=250 resource cancellation (1.19%); n=15,156 evacuation to emergency department (72.37%); n=409 denial to be attended (1.96%); n=2,728 in situ resolution (13.05%); n=966 referral to other professional (4.61%); n=42 unknown (0.20%); priority, n=2891 emergency (13.81%); n=17,606 undelayable emergency (84.07%); n=361 delayable emergency (1.72%); n=82 not urgent (0.39%); n=2 unknown (0.00%); callers 65+ years old 2 times lower likelihood of evacuation compared with younger callers (OR=0.63; 95% CI 0.57-0.69, unadjusted; OR=0.53; 95% CI 0.47-0.59 adjusted); females were more frequently evacuated compared with males (74.53%, p=0.001), & female calls were more frequently resolved in situ (13.40%, p=0.001) |
| **Article** | **Country** | **Crisis Line Description** | **Sample Description** | **Study Time Period** | **Effectiveness Domain(s)** | **Source(s) of Outcome Data** | **Proximity of Outcome Measurement** | **Findings** |
| Table 4b. Distal Evidence on Effectiveness, by Proximity of Outcome Measurement | | | | | | | |  |
| Mishara et al., Study 1,[31] 2016 | USA | Hopeline Network (1-800-SUICIDE), a central toll-free number that transfers calls from anywhere in USA to an accredited participating helpline center (n=91) | n=1,431 adult crisis calls to n=14 centers analyzed (54.8% of all calls) from n=782 crisis helpers; n=4 centers directive model; n=4 centers nondirective model; n=4 all professional centers; n=7 all volunteer centers; n=3 mixed professional/ volunteer centers  Gender, female to male ratio about 3:2;  Age, 47% 18-34 years, 44% 35-54 years, 6% 55-65 years, 1% 65+ years  Suicidal thoughts/ behavior, n=503 suicide crisis calls (35.15%); n=33 attempt in progress (2.3%); n=182 attempt & plan (12.7%); n=288 expressed intent (20.1%) | August 19, 2003 – May 31, 2004 | Caller suicidal thoughts/ behaviors  Caller mood  Caller satisfaction  Caller compliance  Helper response | 11 within-subject ratings by silent monitor on dimensional mood/ state of caller  CCORS  4 factors of helpers’ behaviors/ styles: supportive approach & good contact, active listening, collaborative problem solving, & negative approach; helper’s empathy, respect, & directivity  suicide risk assessment | Change from beginning to end of call | **Caller suicidal thoughts/ behaviors:**  significant decrease in suicidal urgency from beginning to end of call (p<0.001); 76% calls no change suicidal urgency; 16% calls suicidal urgency decreased; 7.8% calls suicidal urgency increased  **Caller mood/ satisfaction:**  at follow-up, 69.2% callers satisfied with help received; 31% not satisfied with help received; female callers improved more frequently than males (18.6% vs 11.8%; p<0.06); CCORS significantly higher in females compared with males (p<0.001)  **Caller compliance:**  42.3% did what they said they would do since initial call; n=39 did not do what they said would do (40.2%); 17.5% no specific contract/ agreement reached  **Helper response:**  overall, no significant differences between volunteers & paid employees on outcomes; volunteers & paid staff with over 140 hours call experience had significantly better outcomes; USA professional centers, 47% calls suicide risk assessment conducted; 26% callers identified as suicidal; 47% calls low empathy; 17% calls low respect; all volunteer centers, 64% calls suicide risk assessment conducted; 34% callers identified as suicidal; significantly higher (more positive) CCORS scores compared with other center types (p<0.01); 27% calls low empathy; 15% calls low respect; mixed centers, 51% calls suicide risk assessment conducted; 24% callers identified as suicidal; 34% calls low empathy; 20% calls low respect; Canada centers, paid staff had no significant differences in intervention styles, changes in suicidal urgency, Psychological Symptom Index, Brasington Indication of Depression, whether contract/ agreement respected compared with volunteers; satisfaction with services significantly higher when paid staff responded to call compared with volunteer staff (78.6% vs 58.3%; p<0.026); more experienced helpers (140+ hours) were less likely to have increase in suicide risk from beginning to end of call (5.4% vs 12.2%), more likely to have improvement in suicide urgency (16.8% vs 14.7%; p<0.02); significantly higher CCORS ratings (p<0.025); more likely for contract/ agreement to be respected (50.1% vs 31.1%; p<0.04) compared with less experienced helpers (less than 140 hours); no difference in satisfaction ratings at follow-up for those with more or less experience |
| Mishara et al., Study 2,[31] 2016 | Canada | Quebec suicide prevention centers | n=1,206 calls analyzed at baseline (73% of monitored calls) from n=5 centers; n=2 paid staff only; n=2 volunteer staff only; n=1 mixed volunteer/paid staff; n=105 contacted for follow-up (8.7% of baseline)  Gender, n=772 females (64%)  Age, NR  Suicidal thoughts/ behavior, 100% with suicidal content | NR | Caller suicidal thoughts/ behaviors  Caller mood  Caller satisfaction  Caller compliance  Helper response | Ratings by silent monitor  Helper Response Scale  Suicidal urgency via 1 item 7-point scale  CCORS  Psychological Symptom Index (abridged) 14-items  Brasington Indication of Depression | Change from beginning to end of call & follow-up call at 1 week |  |
| Kalafat et al.,[32] 2007 | USA | Local crisis hotline telephone line (90.1%), 1-800- SUICIDE for remaining (9.9%) | n=1,617 callers analyzed at baseline (31.3% of eligible callers); n=801 participated in follow-up (49.5% of baseline callers)  Gender, 74.1% female at baseline; 76.4% female at follow-up  Age, M=37.6 years (range 18-85) at follow-up  Suicidal thoughts/ behavior, 100% nonsuicidal crisis | March 2003 – July 2004 | Caller suicidal thoughts  Caller mood  Caller satisfaction  Caller compliance  Caller service utilization  Helper response | POMS-M (confusion, depression, anger, anxiety, helpless, overwhelmed)  hopelessness via 2 questions on 5-point Likert scale  client feedback on call helpfulness (2 questions)  follow-up assessment questions to caller on plan of action/ compliance & service utilization/ compliance | Changes from beginning of call (time 1) to end of call (time 2), & M=13.5 days from baseline assessment (range 1-52 days) (time 3) | **Caller suicidal thoughts:**  n=94 reported suicidal thoughts since call (11.7%); callers with suicidal thoughts at time 3 were significantly more distressed & hopeless at time 3 (p<0.001); significantly more depressed (p<0.001), hopeless, & helpless (p<0.01) at time 1; & more hopeless (p<0.01) at time 2  **Caller mood:**  callers who participated in follow- up were significantly more overwhelmed & received significantly more referrals compared with callers without follow-up (p<0.001); POMS-M, caller distress significantly reduced from time 1 to time 2 & time 2 to time 3 (p<0.001); Significant reduction from time 1 to time 2 & time 2 to time 3 on confusion (p<0.001), depression (p<0.001), anger (p<0.001), anxiety (p<0.001), helpless (p<0.001), & overwhelmed (p<0.001); hopelessness, significant reduction from time 1 to time 2 & time 2 to time 3 (p<0.001)  **Caller satisfaction:**  caller feedback, among n=801 follow-up assessments, callers provided n=1,345 positive responses (listen & talk, warm & caring, dealing with concerns, available & patient, calm down, think clearly/ new perspective) & n=145 negative responses (problems with referral, condescending, not concerned, abrupt, provided unhelpful solutions/ suggestions, not identify problem, call too short, helper asked too many questions) on helpfulness of call  **Caller compliance:**  action plan compliance, among n=801 who participated in follow-up, n=464 developed plans with counselors (57.9%); among those, n=369 recalled the plan (79.5%); among those, n=160 completed all of the plan (43.4%); n=47 completed most of the plan (12.7%); n=54 completed some of the plan (14.6%); n=72 plan still in process (19.5%); n=35 not carried out any of plan (9.5%); n=1 follow through not coded (0.3%); re-contact with center, among n=801 who participated in follow-up, n=186 had another contact (23.2%)  **Caller service utilization:**  among n=392 referred to mental health resource, 33.2% kept or made appointment at time 3  **Helper response:**  n=3 rescues initiated; n=2 followed at time 3; n=1 not followed; referrals, among n=1,617 who participated in baseline; n=969 given new referral (59.9%); among those, n=658 referred to mental health resource (67.9%); n=135 referred back to current therapist/ services (8.3%); among n=801 who participated in follow-up; n=541 given new referral at baseline (67.5%); among those, n=392 referred to mental health resource (72.5%); n=75 referred back to current therapist/ services (9.4%) |
| Gould et al.,[49] 2007; Witte et al.,[50] 2010 | USA | Local crisis hotline telephone line (72.0% of calls), 1-800-SUICIDE for remaining | n=1,085 callers analyzed at baseline (62.4% of suicidal callers); n=380 participated in follow-up (35.3% of baseline)  Gender, 60.6% female at baseline; 69.7% female at follow-up  Age, M=36.1 years (range 18-72) at follow-up  Suicidal thoughts/ behavior, 100% suicidal callers, n=585 had suicide plan; n=88 had taken some action to harm or kill self (8.1%); 22.2% no current plans, actions, or history; 5.7% had plans, actions, & history | March 2003 – July 2004 | Caller suicidal thoughts/ behaviors  Caller mood  Caller satisfaction  Caller compliance  Caller service utilization  Helper response | Suicide risk status assessment via questions to caller, including intent to die (2 questions), hopelessness (2 questions), & psychological pain (2 questions), all rated on 5-point Likert scale  client feedback on call helpfulness (2 questions)  follow-up assessment questions to caller on plan of action/ compliance & service utilization/ compliance | Changes from beginning of call (time 1) to end of call (time 2), & M=13.5 days from baseline assessment (range 1-52 days)* (time 3)  *discrepancy between reports,  range 1-48 days | **Caller suicidal thoughts/ behaviors:**  suicide risk status, significant reductions from time 1 to time 2 on intent to die (p<0.001); hopelessness (p<0.001); & psychological pain (p<0.001); these changes were not modified by suicide risk profile of caller; significant reductions from time 2 to time 3 on psychological pain (p<0.001) & hopelessness (p<0.001); but not intent to die (p>0.05); at time 3, n=164** callers reported any suicidal ideation since time 1 (43.2%); of these, n=28 made suicide plan (17.1%); n=11 made suicide attempt (6.7%; overall 3% of follow-ups); intent to die at time 2 (OR 1.7; 95% CI 1.2-2.3; p<0.001, model 1; OR 1.7; 95% CI 1.2-  2.3; p<0.002, model 2), prior plans to hurt/ kill self (OR 1.6; 95% CI 1.02-2.4; p<0.04), & persistent suicidal thoughts at time 1 (OR 1.6; 95% CI 1.03-2.4 p<0.04) were predictors of any suicidality at time 3 (43.2% of callers); suicidal ideation at time 3 significantly predicted suicide attempt at time 3 (p=0.00); neither resolved plans & preparations nor suicidal desire & ideation predicted subsequent suicidal ideation (p>0.20) or suicide attempts (p>0.20) (model figure 1)  **discrepancy between reports, n=163  **Caller mood:**  suicidal callers who did not complete follow-up were significantly more intent on dying (p<0.001), more hopeless (p<0.001), more likely to be rescued (p<0.001), less likely to be given referral (p<0.001); callers with follow-up were significantly more likely to be female (69.7% vs 55.6%; p<0.001); less likely to have hurt themselves before calling (5.5% vs 9.7%; p=0.02), believe suicide as only option (25.7% vs 34.4%; p=0.004); lower self-reported likelihood of killing self (p=0.00); less hopeless about the future (p=0.01); higher belief they can go on (p<0.0001); more likely history of previous suicide attempt (64.0% vs 55.7%; p=0.01); in sum, follow-up sample less clinically severe compared with those without follow-up data  **Caller satisfaction:**  caller feedback, among n=380 follow-up assessments, callers provided n=668 positive responses (listen & talk, warm & caring, dealing with concerns, available & patient, calm down, think clearly/ new perspective) & n=83 negative responses (problems with referral, condescending, not concerned, abrupt, provided unhelpful solutions/suggestions, not identify problem, call too short, helper asked too many questions) on helpfulness of call; n=44 suicidal callers said call prevented them from harming/ killing self (11.6%)  **Caller compliance:**  action plan compliance, among n=380 follow-up assessments, n=278 developed action plans with helpers (73.2%); among those with action plans, n=60 did not recall plan (21.6%); among those recalling plan, n=102 completed all of the plan (46.8%); n=34 completed most of the plan (15.6%); n=28 completed some of the plan (12.8%); n=24 plan still in process (11.0%); n=26 not carried out any of plan (11.9%); 1.8% follow through not clear  **Caller service utilization:**  follow through with referral, among n=151 given new mental health referral, 35% kept appointment; re-contact with center, among n=380 follow-up assessments, n=107 had another contact (28.2%); n=107 received new referral; n=17 completed referral or setup appointment (15.8%)  **Helper response:**  rescues, n=132 counselors initiated rescue (12.6%); n=54 who took some action to hurt/ kill self no rescue initiated because center unable to identify caller or caller hung up prematurely; rescues initiated more often for callers with current plan compared with those without a plan (19.2% vs 4.9%; p<0.001) & for callers with history of suicide attempt compared with no history (15.2% vs 8.5%; p<0.01); referrals, at baseline, n=506 given referral (46.6%); n=284 referred to mental health resource (56.1%); n=116 referred to current therapist/ services (10.7%); follow-up, n=221 given referral at baseline (58.2%); n=151 to mental health resource (68.3%); n=52 referred to current therapist/ services (13.7%); callers with current plans to hurt/ kill self received fewer referrals compared with those with no plans (44.2% vs 53.0%; p<0.01); & for callers who had taken action to hurt/ kill self compared with those who had not (34.5% vs 49.3%; p=0.01) |
| Gould et al.,[27] 2012 | USA | NSPL (1-800-273-TALK) | n=654 callers who were referred to health care analyzed (52.5% of known eligible) from n=16 centers in n=14 states  Gender, n=431 female (65.9%)  Age, M=38.4 years (SD=12.6)  Suicidal thoughts/ behavior, n=376 suicidal crisis (57.5%) | January 2006 – December 2007 | Caller service utilization  Helper response | Standardized telephone interviews that included Suicide Risk Status, BDI-II, assessment of type of referral(s), mental health care utilization, & barriers to utilization | M=14 days after initial call to the center (SD=10.0, range 3-72 days) | **Caller service utilization:**  43.9% of suicidal callers & 39.2% of crisis callers followed through with referral (41.9% overall); highest rate of follow-through to mental health provider (68.1% vs 64.3%; 66.7% overall); lowest rate of follow-through to new mental health resource (14.3% vs 17.6%; 15.8% overall); of n=151 suicidal callers who did not follow-through with mental health referral, n=38 reported accessing comparable mental health resource (25.2%); of n=111 crisis callers who did not follow-through with mental health referral, n=29 reported accessing comparable mental health resource (26.1%); overall, 51.9% of suicidal callers & 51.2% of crisis callers given a mental health referral utilized mental health services (51.6% overall); utilizing mental health referral not related to demographics, depression, or suicide risk profile; utilization rate in nonsuicidal crisis callers significantly higher among those with insurance compared with those without insurance (59.6% vs 35.6%, OR=0.37; 95% CI 0.19-0.72, p<0.01); utilization rates were significantly higher among those already in treatment compared with those who were not (76.7% vs 26.1%, OR=9.32; 95% CI 5.91-14.70, p<0.0001); 32.9% of those already in treatment utilized a new mental health provider; barriers, n=195 did not utilize mental health resource following call (n=113 suicidal callers; n=82 crisis callers); n=54 not asked about barriers; n=141 asked about barriers; n=75 perceptions about mental health problems (53.2%); n=48 financial barriers (41.2% suicidal callers vs 23.2% crisis callers; OR 2.28; 95% CI 1.08-4.84; p<0.05); n=44 personal barriers (31.2%); n=43 perceptions about mental health services (30.5%); n=43 did not remember referral; n=37 other structural barriers (26.2%); n=11 contacted service & waiting for call back  **Helper response:**  most prevalent type of referral for all callers was to a mental health service (n=403, 61.6%); n=239 referred to phone services (36.5%), n=99 referred to social services (15.1%), n=98 referred to counseling by non-mental health professional (15%), n=70 referred to emergency services (10.7%); females were more likely than males to receive mental health service referral (66.7% vs 54.9%; OR=1.72; 95% CI 1.11-2.68, p<0.05); suicidal callers were more likely to receive referral to emergency services compared with crisis callers (13.3% vs 7.2%; OR=1.77; 95% CI 2.05-2.96, p<0.05); all other referral service types not significantly different between groups |
| Fukkink & Hermanns,[12] 2009 | Amsterdam | Dutch Kindertelefoon chat & phone services | n=902 participants with complete data analyzed (1.3% of those who contacted service); n=339 chatters; n=563 callers; n=223 analyzed at follow-up (24.7% of baseline); n=119 chatters (35.1% of baseline); n=94 callers (16.7% of baseline)  Gender, n=272 female chatters (80%); n=401 female callers (71%)  Age, chatters, M=13.8 years (SD=2.0); callers, M=12.0 years old (SD=2.3)  Suicidal thoughts/ behavior, NR | May – August 2006 | Caller/ chatter mood  Caller/ chatter satisfaction | Well-being via 9-point scale answer to 1 question how you feel at the moment (time 1, time 2, & time 3)  perceived burden of the problem via 1 question 9-point scale (time 1, time 2, & time 3)  satisfaction with the conversation via 8 questions each on a 9-point scale (time 2 only) | Change from beginning (time 1) to end of contact (time 2) & at 1-month follow-up (time 3) | **Caller/ chatter mood/ satisfaction:**  significant increase in sense of well-being from time 1 to time 2 as well as time 1 to time 3 (both p<0.001); significant decrease in burden of problem from time 1 to time 2 as well as from time 1 to time 3 (both p<0.001); results were significantly more favorable for chat group compared with phone group for improved well-being (p=0.02) & burden of problem (p<0.001) at time 2, positive effect applied equally to both groups at time 3 (p not significant); chat participants reported feeling supported, being taken seriously, made to feel at ease, comprehensible, & not disorganized significantly more compared with telephone participants (all p<0.001) |
| Gould et al.,[51] 2018 | USA | NSPL (1-800-273-TALK) | n=550 callers with at least n=1 follow- up call interviewed (23.7% of callers with clinical follow-up), followed by n=41 crisis counselors from n=6 centers  Gender, n=348 female (63.3%)  Age, n=131 18-24 years old (23.8%); n=137 25-34 years old (24.9%); n=106 35-44 years old (19.3%); n=115 45-54 years old (20.9%); n=61 55+ years old (11.1%)  Suicidal thoughts/ behavior, n=293 wish to die (53.6%); n=274 suicide plans (50.0%); n=187 suicidal behavior (34.2%) | April 2009 – September 2011 | Caller suicidal thoughts/ behaviors  Helper response | Self-report counselor questionnaire on follow-up activities developed for the study  standardized telephone interview with follow-up clients developed for study | Between 6 – 12 weeks after initial call to center (M=57.3 days after initial call; SD 12.0, range 42–150 days; M=36.9 days after most recent follow-up call; SD 20.3, range 0–142 days); follow-up structure, n=106 (19.3%) follow-up still ongoing at time of interview; M=2.4 follow-up calls (SD 1.6) prior to interview; M=51.4 minutes of follow-up calls (SD 45.4, range 2-289 minutes); M=13 days from 1st to last follow-up call (SD 15.4); M=7.4 days between crisis call & 1st follow-up (SD 7.5) | **Caller suicidal thoughts/ behaviors:**  clients’ perceptions of care, 79.6% reported intervention stopped them from killing themselves (53.8% a lot; 25.8% a little; 20.4% not at all); 90.6% reported intervention kept them safe (59.6% a lot; 31.0% a little; 9.5% not at all); n=11 (2.0%) did not provide feedback because could not remember having a follow-up call; n=0 reported the follow-up calls made things worse; stopped from killing self significant associations, Hispanic ethnicity (OR 1.99; 95% CI 1.14-3.46; p=0.015); as well as <high school education (OR 1.84; 95% CI 1.26-2.70; p=0.002); adult homelessness (OR 1.86; 95% CI 1.25-2.76; p=0.002); baseline suicide risk score (OR 1.16; 95% CI 1.026-1.302; p=0.017); lifetime attempt (OR 1.46; 95% CI 1.05-2.03; p=0.026); number of follow-up calls (p<0.001); number of minutes of follow-up calls (OR=1.008; 95% CI 1.002-1.013; p=0.005); days between 1^st^ & last follow-up (OR 1.024; 95% CI 1.011-1.037;  p=0.003); discussed safe/ no use alcohol/ drugs (OR 1.51; 95% CI 1.01-2.26; p=0.005); social contacts/ settings to use as distractors (OR 2.14; 95% CI 1.25-3.67; p=0.005); social contacts to call for help (OR 2.27; 95% CI 1.36-3.77; p=0.002); & explored reasons for dying (OR 1.72; 95% CI 1.15-2.56; p=0.008);  kept safe significant associations, female (OR 1.54; 95% CI 1.09-2.19; p=0.014); as well as age (OR 1.02; 95% CI 1.003-1.031; p=0.016); Hispanic (OR 2.30; 95% CI 1.27-4.18; p=0.006); <high school education (OR 1.91; 95% CI 1.28-2.86; p=0.002); adult homelessness (OR 2.02; 95% CI 1.33-3.07; p=0.001); lifetime attempt (OR 1.61; 95% CI 1.14-2.27; p=0.007); number of follow-up calls (p<0.001); number of minutes of follow-up calls (OR=1.010; 95% CI 1.004-1.016; p=0.001); days between 1^st^ & last follow-up (OR 1.023; 95% CI 1.009-1.036; p=0.001); discussed safe/ no use alcohol/ drugs (OR 1.65; 95% CI 1.07-2.50; p=0.019); social contacts/ settings to use as distractors (OR 2.58; 95% CI 1.49-4.47; p=0.001); social contacts to call for help (OR 2.95; 95% CI 1.75-4.96;  p<0.001); discussed triggers to suicidality (OR 2.30; 95% CI 1.33-3.97; p=0.003); discussed warning signs (OR 2.69; 95% CI 1.55-4.66; p=0.0004); explored reasons for dying (OR 2.41; 95% CI 1.58-3.66; p<0.001); explored ambivalence about life/ death (OR 1.69; 95% CI 1.15-2.48; p=0.008)  **Helper response:**  counselor follow-up activity, n=527 discussed coping strategies (95.8%); n=526 offered emotional support (95.6%); n=487 social contacts/ settings as distractors (88.5%); n=481 social contacts to call for help (87.5%); n=475 discussed past survival skills (86.4%); n=472 discussed triggers to suicidality (85.8%); n=470 discussed warning signs (85.5%); n=424 explored reasons for living (77.1%); n=336 discussed safe/ no use of alcohol/ drugs (61.1%); n=324 discussed making environment safe (58.9%); n=303 explored reasons for dying (55.1%); n=272 explored ambivalence about life/ death (49.5%) |
| Jianlin,[52] 1995 | China | Hotline for Mental Health in Shanghai | n=8,214 calls analyzed with complete records (56% of all calls); n=71 cases at follow-up (0.8% of baseline)  Gender, n=3,138 female (38.2%)  Age, n=240 15 years (2.9%); n=3,584 16-25 years (43.6%); n=2,301 26-35 years (28.0%); n=747 36-45 years (9.1%); n=203 46-55* years (2.5%); n=92 56 years (1.1%)  *Interpreted from typo in article Table 1  Suicidal thoughts/ behavior, n=117 considering suicide attempt (1.4%) | November 1990 – October 1992 | Caller suicide  Caller mood  Caller service utilization  Helper response | NR | During call, follow-up after 3 months | **Caller suicide:**  n=1 suicide (1%)  **Caller mood:**  follow-up at 3 months, n=58 improved & felt better (77%); n=9 no improvement (12%)  **Caller service utilization:**  n=3 hospitalized for treatment of suicide risk (4%)  **Helper response:**  psychosocial approaches used, n=2,642 explanation (general) (32.2%); n=2,043 explanation (directive) (24.9%); n=1,249 coping skills training (15.8%); n=1,071 catharsis & ventilation (13.0%); n=900 empathy (11.0%); n=575 education on mental health (7.0%); n=476 cognitive & attitude modification (5.8%); n=385 social support (4.7%); n=291 social skills training (3.5%); n=110 environmental management suggestion (1.3%); n=52 suggestion (0.6%); n=394 other psychosocial intervention (4.8%); n=65 medication guidance (0.8%); n=332 referral to other agencies (4.0%); n=176 unrecorded (2.1%) |
| Mishara et al.,[16] 2005 | Canada | Suicide Action Montreal | n=131* family & friends of suicidal men analyzed (36.9% of eligible); Information session, n=71 (52.2% of invited); n=36 randomly selected for analysis; information session + follow-up, n=36 analyzed (32.1% of invited); rapid referral, n=23 analyzed (40.4% of invited); telephone support, n=35 analyzed (46.7% of invited)  *discrepancy in results, n=130  Gender, 81.7% female (callers)  Age, M=44.2 years (callers)  Suicidal thoughts/ behavior of man caller concerned about, 100% had already attempted suicide at least once | February 14, 2000 – January 15, 2002 | Suicidal thoughts/ behaviors of man caller concerned about  Service utilization of suicidal man  Mood of suicidal man  Caller satisfaction | 2 questionnaires completed by caller concerned about suicidal man  coping mechanisms via Ways of Coping Questionnaire (4-point Likert scale)  knowledge & utilization of resources via open-ended questions & satisfaction via Likert scale, also asked concerning use of resources by suicidal man plus additional question regarding keeping appointments  social relationships via Quebec Health Survey, also asked concerning social relationships of suicidal man  communications with suicidal man via 10 questions developed for study  psychological distress via 14 items on 4-point Likert scale from Quebec Health Survey, plus 4 additional questions  suicidal behaviors via 9 questions  communication with friend/ family member via yes/ no question & follow-up question on 4-point Likert scale if yes  alcohol consumption via 5 questions from Quebec Health Survey  satisfaction & perceived usefulness via questions on 4-point Likert scale  questionnaire rating family/ friend participant completed by persons conducting programs | Post-test 2 months after receiving services & follow-up 6 months after 1^st^ evaluation | **Suicidal thoughts/ behaviors of man caller concerned about:**  participants reported suicidal men were significantly less likely to have seriously considered suicide after participation (post-test p<0.001*; 6 months p<0.01*), less frequently attempted suicide in previous 2 months (post-test p<0.02*; 6 months p<0.001*)  **Service utilization of suicidal man:**  Suicidal men used significantly fewer resources at follow-up (85.8% pre-test; 48.4% post-test; 23.0% 6 months; p<0.01) & significantly fewer number of resources proposed at follow-up (p<0.02)  **Mood of suicidal man:**  fewer symptoms of depression than in 2 months prior to participation (post-test p<0.001**; 6 months p<0.001**)  **Caller satisfaction:**  n=224 declined to participate in program; 20.8% unavailable to attend sessions; 18.2% did not need additional help after call; 10.6% program proposed did not meet needs; 7.6% program involved too much effort; 3.6% wanted to keep distance & avoid getting too involved with suicidal man; overall, M=3.56/4 satisfied with help (SD 0.67); helpful to understand suicide, better communication & ability to help; not helpful increasing use of other resources or decreasing suicidal man’s use of alcohol/ drugs, changing social activities; No significant differences between programs in these changes; Participants in programs experienced less psychological distress (p<0.001), less disruptive to family, professional, social life (p<0.01) comparing pre-test with post-test (& maintained follow-up); Participants used significantly more coping mechanisms (p=0.002), more frequently (p=0.011), more positive (p=0.001) more frequently (p=0.004) to deal with stress comparing pre-test with post-test (no 6 month data available); programs did not results in significant increase in knowledge/ use of resources by participants or suicidal men  **discrepancy in report, it appears the “>” symbol was used in error (should be “<”)  family sessions abandoned due to lack of participation (n=1 out of n=18 approached accepted participation); information Session, M=3.3/ 4 actively participated/ discussion; M=3.46/ 4 overall satisfaction (SD 0.74); telephone follow-up, M=3.65/ 4 overall satisfaction (SD 0.49); 88.5% contacted as planned; 90% had been in contact with suicidal man between session & telephone contact; 81.5% practices suggested interaction; 32% had questions for telephone helper; 55.6% asked for specific suggestions (what to do/ referral); rapid referral, M=3.29/ 4 overall satisfaction (SD 0.85); 19% suicidal men contacted agencies to which they were referred; participants proposed significantly fewer resources compared with other programs (p<0.001); telephone support: M=3.73/ 4 overall satisfaction (SD 0.58); participants in telephone support were significantly more satisfied with length of intervention (p<0.02), better met their needs (p<0.05), more helpful in understand mental health problems (p<0.01), improving communication (p<0.01), increasing use of other resources (p<0.05), & proposed significantly more external resources to the suicidal man compared with other programs; suicidal men spent significantly less time alone (p<0.01) & significant increase in social contacts (p<0.05) comparing pre-test with post-test (& maintained follow-up) |
| Britton et al.,[19] 2016 | USA | VCL | n=13,444 callers analyzed (64% of eligible referrals); n=12,265 prior VHA use (past 5 years) (91%); n=1,179 no prior VHA use (past 5 years) (9%)  Gender, Prior VHA use, 12% female; No prior VHA use, 11% female  Age, Prior VHA use, M=48 years (SD=13.45); No prior VHA use, M=42 years (SD=14.31)  Suicidal thoughts/ behavior, primary diagnosis suicide & suicidal ideation, prior VHA use, n=476 (3.92%); no prior VHA use, n=72 (6.15%) | Calendar year 2010 | Caller service utilization  Helper response | VHA service utilization via clinical records | Mental health & inpatient VHA services related to VCL call accessed within 180 days of referral | **Caller service utilization:**  VCL is most frequently used by Veterans engaged in VHA care; callers with prior VHA use more likely to receive mental health or substance use disorder diagnoses (all p<0.01); callers without prior VHA use more likely to received suicide-related diagnoses (p=0.002) & “other” mental health-related diagnoses & diagnoses influencing health status (all p<0.01); callers with prior VHA use more likely to be middle aged (p<0.0001), callers without prior use more likely to be younger (p<0.0001); 91% with prior VHA use & 71% without prior VHA use presented for in-person VHA care within 7 days of referral; callers with prior VHA use more likely to present for care same day as referral (44% vs 25%, p<0.0001); callers without prior VHA care more likely to present for care after 15 days (p<0.0001); among callers with suicide-related diagnoses, 97% with prior use made contact within 7 days of call compared with 96% without prior use (p>0.05); callers with prior use had greater mean number of outpatient mental health encounters within 30 days of referral (M=4.4 days, SD=5.4 vs M=3.6 days, SD=4.7)  **Helper response:**  n=21,130 calls with referrals made (20.6%) |
| Leenaars & Lester,[17] 1995 | Canada | Crisis centers in each Canadian province | n=12 regions  Gender, Age, Suicidal thoughts/ behavior, NR | 1985 - 1991 | Suicides in region | Number of suicide prevention & crisis centers obtained from Canadian Council of Crisis Centres | Absolute & percentage change in suicide rates 1985-1991 | **Suicides in region:**  generally negative correlations, indicating that the more crisis services present in a province, the more likely suicide rate decreased (results not significant) |
| Leenaars & Lester, Study 1,[18] 2004 | Canada | n=89 Crisis centers in Canadian provinces in 1985 | n=12 regions  Gender, Age, Suicidal thoughts/ behavior, NR | 1985 - 1989 | Suicides in region | Number of suicide prevention & crisis centers obtained from Canadian Council of Crisis Centres | Age-adjusted absolute & percentage change in suicide rates 1985-1989 | **Suicides in region:**  1985-1989, generally negative correlations, indicating that the more crisis services present in a province, the more likely suicide rate decreased (results not significant); 1994-1998, generally negative correlations, indicating that the more crisis services present in a province, the more likely suicide rate decreased; centers/ capita change in suicide rates -0.65 (p<0.05), % change -0.59 (p<0.10);  centers/ area change in suicide rates -0.61 (p<0.10), % change -0.59 (p<0.10) |
| Leenaars & Lester, Study 2,[18] 2004 | Canada | n=195 Crisis centers in Canadian provinces in 1994 | n=12 regions  Gender, Age, Suicidal thoughts/ behavior, NR | 1994 - 1998 | Suicides in region | Number of suicide prevention & crisis centers obtained from Canadian Association for Suicide Prevention | Age-adjusted absolute & percentage change in suicide rates 1994-1998 |  |
| Chan et al.,[20] 2018 | Hong Kong | The CCS telephone helpline in Hong Kong by trained staff or volunteers | n=106,583 older adult (65+ years) users of telephone helpline; n=563,346 person-years analyzed  Gender, n=69,957 females (65.6%)  Age, M=77 years, n=39,790 65-74 years (37.3%); n=53,186 75-84 years (49.9%); n=13,607 85+ years (12.8%)  Suicidal thoughts/ behavior, NR | January 1, 2012 – December 31, 2015 | Caller suicides  Suicides in region | Sociodemographic data from service’s computerized system which users provided when registering to service  suicide mortality status from the Coroner’s Court matched against crisis callers using unique Hong Kong Identity Card Number  population suicide statistics from Hong Kong Census & Statistics Department | Suicide statistics from 2012 – 2015 | **Suicides:**  n=1,006 older (65+ years old) adult suicides in Hong Kong; n=145 were users of helpline (14.4% of all known suicides; 11.3% of male suicides; 19.8% of female suicides); suicide rate among helpline users far higher than general Hong Kong older adult population (86.3 vs 32.6 per 100,000; IRR=2.6); significant predictors of suicide, older age, 75-84 years old (HR=1.84; 95% CI 1.26-2.69); 85+ years old (HR=3.64; 95% CI 2.14-6.17); male (HR=2.45; 95% CI 1.74-3.43); living alone (HR=1.70; 95% CI 1.20-2.42); self-reported mental illness (HR=6.24; 95% CI 3.67-10.58); skeletal system diseases (HR=0.57; 95% CI 0.37- 0.90); brain & nervous system diseases (HR=0.47; 95% CI 0.26-0.85); 60% of helpline user suicides occurred within 5 years of use of service |
| Pil et al.,[14] 2013 | Belgium | Flemish suicide helpline De Zelfmoordlijn (phone & chat services) | n=3,785 unique users contacted helpline seeking personal help  Gender, n=2,418 females (64%); n=1,840 female telephone users; n=578 female chat users  Age, M=37 years (range 12-91); telephone M=42 years; chat M=21 years  Suicidal thoughts/ behavior, 48% of female telephone service  users & 59% of female chat service users had moderate to  strong suicidal thoughts;  46% of male telephone service users & 55% of male chat service users had moderate to strong suicidal  thoughts | 2011 | Predicted suicides  Predicted suicide attempts  Predicted QALYs  Predicted cost-savings | Incremental cost-effectiveness ratio via predicted costs over 10 years divided by the predicted net effects in QALYs  age- & gender-dependent transition probabilities derived from published literature, Flemish databases, & Flemish Centre of Suicide Prevention  costs via Belgian hospitalization & general practitioner consultation data, & American study estimating cost of suicide (ambulance transport, medical examiner costs, emergency department, inpatient hospitalization, nursing home costs) converted using purchasing power parity; lost productivity measured via absenteeism, unemployment, retirement; costs of running helpline  QALY measured via EQ-5D index utilities weighted utility of suicidal thoughts | Prediction of life events over a 10-year period comparing scenarios with the helpline present vs helpline absent; 1-year cycles in which 1 of 6 transition states were allowed each year (initial state, first attempt, follow-up, re-attempt, suicide, & death from other causes) | **Predicted suicides/ attempts:**  over n=10 years, telephone/ chat service could avoid 36% of suicide attempts (n=205 attempts & n=33 suicides)  **Predicted QALYs:**  males, 0.063 QALYs (95%CI 0.030-0.097) gained by telephone users & 0.035 QALYs (95% CI -0.026-0.096) gained by chat users; females, 0.019 QALYs (95% CI -0.015-0.052) gained by telephone users & neutral for chat users (-0.005; 95% CI -0.071-0.062)  **Predicted cost-savings:**  €2382 (95% CI 1953–2859) & €2282 (95% CI 1855–2758) in male phone & chat users, respectively; €2171 (95% CI 1735–2664) & €2458 (95% CI 1945–3025) in female phone & chat users, respectively; an investment of €218,899 saved €1,452,022 for national health insurance (€1,188,519 through telephone service & €263,503 through chat service) |
| **Article** | **Country** | **Crisis Line Description** | **Sample Description** | **Study Time Period** | **Effectiveness Domain(s)** | **Source(s) of Outcome Data** | **Proximity of Outcome Measurement** | **Findings** |
| Table 4c. Unclear Proximity of Outcome Measurement Evidence on Effectiveness | | | | | | | | |
| Daigle & Mishara,[53] 1995 | Canada | Suicide-Action Montreal & Carrefour Intervention Suicide in Quebec*, French-speaking centers, delivered by volunteers  *Note: Quebec center is open only Mondays to Saturdays, 8a-12a, with recording system at other times | n=263 callers observed with n=110 helpers (42.6% of all calls)  Gender, 59% female  Age, 13-72 years (M=35, SD=12)  Suicidal thoughts/ behavior, 100% suicidal | 1988 (Suicide-Action Montreal only) & 1990 (both centers) | Helper response | 20-category Helper’s Response List rating instrument developed by researchers & used by observers of crisis calls | During call | **Caller suicidal thoughts/ behaviors:**  suicide urgency, mean decrease from beginning to end of call (M=0.40; SD=0.78; p<0.001); suicide urgency decreased in n=138 calls (27%), increased in n=2 calls (1%), & remained the same for majority of calls; suicide urgency decreased significantly more frequently among nonchronic callers compared with chronic callers (35% vs 24% p<0.01; nonchronic, M=0.51; SD=0.85 vs chronic M=0.51; SD=0.85; p<0.05); n=3 callers attempted suicide after contact with center (1%)  **Caller mood:**  depressive mood, mean decrease from beginning to end of call (M=0.16; SD=0.45, p<0.001); depression decreased in n=85 of n=613 calls (14%), remained the same for majority of calls (85%), & increased in n=3 calls (1%); depression decreased in 12% of chronic callers & 17% of nonchronic callers (not significant)  **Caller compliance:**  54% of contracts upheld as indicated in follow-up contacts; 14% of contracts made were not upheld; female callers significantly more likely to respect contracts compared with male callers (p-value not reported); 14% calls terminated prematurely by caller  **Helper response:**  n=391 calls classified as Rogerian (nondirective) style; n=226 directive style; Rogerian style associated with older callers & less at risk for suicide, fewer chronic or intoxicated callers; Rogerian style more likely when callers are medicated or report previous suicide attempt; Rogerian style significantly more decreases in depression (16% vs 10%; p<0.01), & more likely to make contract with caller (72% vs 61%; p<0.01); Rogerian techniques related to significantly greater reductions in suicidal urgency (p<0.05), & significantly greater likelihood of making a contract (p<0.05) among nonchronic callers compared with chronic callers; contract made with callers in 68% of calls; no contract made in 17% of calls; contracts made more frequently with chronic callers compared with nonchronic callers (79% vs 49%; p<0.001) |
| Mishara & Daigle,[54] 1997 | Canada | Suicide-Action Montreal & Carrefour Intervention Suicide in Quebec*, French-speaking centers, delivered by volunteers  *Note: Quebec center is open only Mondays to Saturdays, 8a-12a, with recording system at other times | n=263 callers observed with n=110 helpers (42.6% of all calls)  Gender, 59% female  Age, 13-72 years (M=35, SD=12)  Suicidal thoughts/ behavior, 100% suicidal | 1988 (Suicide-Action Montreal only) & 1990 (both centers) | Caller suicidal thoughts/ behaviors  Caller mood  Caller compliance  Helper response | 20-category Helper’s Response List rating instrument developed by researchers & used by observers of crisis calls  observer ratings on depressive mood, measured by single-item 5-point Brasington Depression Scale  telephone volunteer ratings on suicidal urgency, measured by single-item 9-point Suicide Urgency Scale on the probability of caller making a suicide attempt in the next 2 days  follow-up information on upholding a contract & not attempting suicide measured by whether contract made with the client & subsequent follow-up calls based on written records in the dossier of each caller | Change from beginning to end of call; subsequent follow-up calls (time from initial call not reported) |  |
| Chavan et al.,[55] 2012 | India | 24-hour suicide prevention helpline by counselors (psychologists) through the Department of Psychiatry, GMCH, Chandigarh | n=3,273 calls analyzed  Gender, n=1,115 females (34.07%)  Age, n=124 <20 years (3.79%); n=946 20-29 years (28.90%); n=898 30-39 years (27.44%); n=586 40-49 years (17.90%); n=719 >49 years (21.97%)  Suicidal thoughts/ behavior,  n=80 callers diagnosis of intentional self-harm (2.44%) | August 2004 – April 2009 | Suicides in region  Caller service utilization  Helper response | Counselor-recorded responses in registers maintained in helpline  suicides in city of Chandigarh (source not described) | Outcome at end of call; hospital visit records (timeframe not described) | **Suicides in region:**  n=130 in 2003; n=75 in 2004; n=89 in 2005; n=80 in 2006; n=82 in 2007; n=83 in 2008; n=75 in 2009; n=71 in 2010; n=105 in 2011  **Caller service utilization:**  n=183 seen in psychiatry OPD (16.43% of those referred); n=72 brought in by CIT & admitted to ward; n=39 home visits; n=26 admitted through helpline after receiving calls from general public (n=18 rehabilitated at homes; n=8 shelter homes); n=128 call backs  **Helper response:**  n=1,114 referred to psychiatry OPD; n=312 referred to other health care facility; n=1054 only counseling done; n=459 referred to CIT; n=334 conference consultation; n=973 follow-ups |

*Note*. USA = United States of America, UK = United Kingdom; NR = Not reported; MINI = Mini-International Neuropsychiatric Interview; CCORS = Crisis Call Outcome Rating Scale; SD = Standard Deviation; SI = Suicidal ideation; VCL = Veterans Crisis Line; NSPL = National Suicide Prevention Lifeline; EPES = Empresa Publica de Emergencias Sanitarias; M = Mean; GMCH = Government Medical College & Hospital; CCS = Care & Call Service; SPSCMP = Suicide Prevention & School Crisis Management Program; DCPS = Dade County Public Schools; VHA = Veterans Health Administration; BDI-II = Beck Depression Inventory; SPC = Suicide Prevention Coordinator; RRR = Relative Risk Ratios; OPD = Outpatient Department; CIT = Crisis Intervention Team; IRR = Incident Rate Ratios; HR = Hazard Ratios; SMST = Silent Monitoring Study of Telephone helplines; POMS-M = Profile of Mood States Modified; MS = Median Score; IQR = Interquartile Range; TASP = Transit Authority Suicide Prevention; ED-NOS = Eating Disorder Not Otherwise Specified; BED = Binge Eating Disorder; AN = Anorexia Nervosa; BN = Bulimia Nervosa; RCT = Randomized Controlled Trial; ASIST = Applied Suicide Intervention Skills Training; SI = Suicidal Ideation; SA = Suicide Attempt; QALY = Quality-Adjusted Life Years
